# Kinesin-8 and Dis1/TOG collaborate to limit spindle elongation from prophase to anaphase A for proper chromosome segregation in fission yeast

**DOI:** 10.1242/jcs.232306

**Published:** 2019-09-15

**Authors:** Corinne Pinder, Yuzy Matsuo, Sebastian P. Maurer, Takashi Toda

**Affiliations:** 1 Cell Regulation Laboratory, The Francis Crick Institute, 1 Midland Road, London NW1 1AT, UK; 2 Division of Biological and Life Sciences, Graduate School of Integrated Sciences for Life, Hiroshima University, 1-3-1 Kagamiyama, Higashi-Hiroshima, Hiroshima 739-8530, Japan; 3 Synthetic and Systems Biochemistry of the Microtubule Cytoskeleton Laboratory, The Francis Crick Institute, 1 Midland Road, London NW1 1AT, UK; 4 Centre for Genomic Regulation (CRG), Barcelona Institute of Science and Technology (BIST), Dr. Aiguader 88, 08003 Barcelona, Spain; 5 Universitat Pompeu Fabra (UPF), 08002 Barcelona, Spain; 6 Hiroshima Research Center for Healthy Aging (HiHA), Hiroshima University, 1-3-1 Kagamiyama, Higashi-Hiroshima, Hiroshima 739-8530, Japan

**Keywords:** Fission yeast, Kinesin-8, XMAP215/TOG, Spindle elongation, Microtubules

## Abstract

High-fidelity chromosome segregation relies on proper microtubule regulation. Kinesin-8 has been shown to destabilise microtubules to reduce metaphase spindle length and chromosome movements in multiple species. XMAP215/chTOG polymerases catalyse microtubule growth for spindle assembly, elongation and kinetochore-microtubule attachment. Understanding of their biochemical activity has advanced, but little work directly addresses the functionality and interplay of these conserved factors. We utilised the synthetic lethality of fission yeast kinesin-8 (Klp5-Klp6) and XMAP215/chTOG (Dis1) to study their individual and overlapping roles. We found that the non-motor kinesin-8 tailbox is essential for mitotic function; mutation compromises plus-end-directed processivity. Klp5-Klp6 induces catastrophes to control microtubule length and, surprisingly, Dis1 collaborates with kinesin-8 to slow spindle elongation. Together, they enforce a maximum spindle length for a viable metaphase–anaphase transition and limit elongation during anaphase A to prevent lagging chromatids. Our work provides mechanistic insight into how kinesin-8 negatively regulates microtubules and how this functionally overlaps with Dis1 and highlights the importance of spindle length control in mitosis.

## Abstract (precis)

**Summary:** The kinesin-8 Klp5-Klp6 relies on tailbox motifs for processivity, thereby inducing catastrophe and destabilising MTs. Klp5-Klp6 and Dis1/TOG, acting in concert, impose an upper spindle length for viable chromosome segregation in fission yeast.

## INTRODUCTION

The mitotic spindle is a complex, macromolecular structure that, subject to precise control, orchestrates the accurate segregation of chromosomes; aberrations in this process can lead to aneuploidy, a risk factor for cancer and various human diseases (Zhang et al., 2010; Hanahan and Weinberg, 2011). Microtubules (MTs) constitute the core of the spindle. They are dynamic, polar polymers (Weisenberg et al., 1968; Mitchison and Kirschner, 1984; Mitchison, 1993) that form a physical scaffold for the mitotic apparatus and recruit a vast array of MT-associated proteins (MAPs) to regulate their length and behaviour.

Among this ensemble of MAPs, the kinesin-8 family of motor proteins are remarkable in that they translocate along MTs towards plus ends (Gupta et al., 2006; Mayr et al., 2007; Grissom et al., 2009; Lee et al., 2010; Erent et al., 2012) and promote MT destabilisation (Gupta et al., 2006; Varga et al., 2006; Mayr et al., 2007; Unsworth et al., 2008; Tischer et al., 2009). Kinesin-8 processivity is high (Varga et al., 2006; Mayr et al., 2011; Möckel et al., 2017; McHugh et al., 2018), facilitating their accumulation at plus ends for MT length control (Varga et al., 2006; Gupta et al., 2006; Stumpff et al., 2008; Tischer et al., 2009), which is a major but not sole aspect of their mitotic function in addition to such roles as kinetochore (KT)-MT attachment (Garcia et al., 2002b; Mary et al., 2015; Klemm et al., 2018; Edzuka and Goshima, 2019).

Although kinesin-8 proteins are conserved from yeast to humans, their structure, function and biochemical activity have diverged. Three different homodimers constitute the kinesin-8 repertoire in human cells: Kif19A depolymerises MTs in ciliary length control (Niwa et al., 2012), Kif18B negatively regulates astral MT length and number (Lee et al., 2010; Stout et al., 2011; Tanenbaum et al., 2011) and Kif18A reduces spindle length (Mayr et al., 2007, 2011; Stumpff et al., 2008) and suppresses KT-MT dynamics for proper chromosome congression (Stumpff et al., 2008; Mayr et al., 2011; Weaver et al., 2011).

*Saccharomyces* *cerevisiae* expresses a single homodimeric kinesin-8, Kip3, that is required for KT clustering in metaphase (Wargacki et al., 2010), reducing spindle length during anaphase (Straight et al., 1998; Rizk et al., 2014) and sustaining depolymerisation of interpolar MTs (iMTs) for spindle disassembly (Woodruff et al., 2010) through its MT destabilising and sliding activities.

*Schizosaccharomyces* *pombe* is unique in that its two kinesin-8 proteins (kinesin-like proteins 5 and 6) form a heterodimer Klp5-Klp6 (West et al., 2001; Garcia et al., 2002b; Li and Chang, 2003; Unsworth et al., 2008). The complex localises along the length of the early spindle, interacts with KTs in metaphase and appears to be restricted to the spindle midzone during anaphase B (West et al., 2001; Garcia et al., 2002b), yet its exact molecular function at these spindle locations requires further investigation. It is known that Klp5-Klp6 reduces metaphase spindle length (Garcia et al., 2002b), partly by antagonising spindle stability generated by the MT crosslinker Ase1 (Syrovatkina et al., 2013). Klp5-Klp6 also contributes to chromosome congression to the spindle centre (Garcia et al., 2002b; Mary et al., 2015), at least in part by destabilisation of KT-MT attachment that generates pulling forces (Mary et al., 2015; Klemm et al., 2018). Furthermore, Klp5-Klp6 limits the appearance of lagging chromatids during anaphase (Garcia et al., 2002b; Gergely et al., 2016) but how this is executed is not known. Both proteins bind protein phosphatase 1 (PP1), an interaction required for efficient spindle assembly checkpoint (SAC) silencing (Meadows et al., 2011). It remains to be seen whether Klp5 and Klp6 are, like Kif18A, substrates of PP1 and whether phosphorylation regulates accumulation at MT plus ends (De Wever et al., 2014).

Furthermore, the effect of Klp5-Klp6 on dynamic MTs has not been studied *in vitro*. In contrast, extensive studies of Kip3 have shown that the budding yeast kinesin-8 directly depolymerises MTs (Varga et al., 2006; Gupta et al., 2006). Kif18A, Kif18B and Kip3 use secondary MT-binding sites in the tail domain for efficient accumulation at KT-MT plus ends (Mayr et al., 2011; Stumpff et al., 2011; Su et al., 2011; McHugh et al., 2018) and some could undergo auto-inhibition.

The conserved XMAP215/chTOG/Dis1 (tumour overexpressed gene, TOG) family of MT polymerases is also a key set of mitotic MAPs. These plus-tip-tracking MAPs polymerise MTs by binding to MTs and capturing free tubulin for incorporation (Al-Bassam et al., 2006; Widlund et al., 2011; Ayaz et al., 2012; 2014; Matsuo et al., 2016; Nithianantham et al., 2018; Cook et al., 2019). However, it seems that in the absence of free tubulin some TOGs can also catalyse MT depolymerisation *in vitro* (Brouhard et al., 2008; Roostalu et al., 2015; Matsuo et al., 2016).

*S. pombe* expresses two nonessential TOG genes, *alp14* (also known as *mtc1*) and *dis1*. Although both Alp14 and Dis1 are verified MT polymerases (Al-Bassam et al., 2012; Matsuo et al., 2016; Nithianantham et al., 2018; Cook et al., 2019) and double deletions are lethal (Garcia et al., 2001; Nakaseko et al., 2001), these TOG are not simply paralogues. Alp14 forms a complex with Alp7/transforming acidic coiled-coil (TACC) (Sato et al., 2004) and is required for bipolar spindle formation through interaction with γ-tubulin at the spindle pole body (SPB) (Garcia et al., 2001; Tang et al., 2014). In contrast, Dis1 is not required for spindle assembly (Ohkura et al., 1988; Nakaseko et al., 2001). Dis1 interacts with Mal3/EB1 and the outer KT Ndc80 complex where it couples chromosome movements to MT dynamics until metaphase (Hsu and Toda, 2011; Matsuo et al., 2016) and relocalises to spindle MTs during anaphase; this redistribution is controlled by CDK1-mediated phosphorylation and subsequent dephosphorylation (Aoki et al., 2006). Cells lacking Dis1 are cold sensitive and cannot progress through mitosis because sister chromatids remain non-disjoined on dysfunctional spindles (Ohkura et al., 1988; Hsu and Toda, 2011). Intriguingly, deletions of either *alp14* or *dis1* MT polymerases are synthetically lethal with the MT-destabilising *klp5* and *klp6* deletions (West et al., 2002; Garcia et al., 2002a), but the reasons for this remain to be determined.

We sought to address the aforementioned issues using a variety of approaches. We identified a role for the Klp5-Klp6 non-motor tailbox (TB) motifs in kinesin processivity, which is important for overall mitotic function. We demonstrate that these fission yeast kinesin-8 proteins control MT length by inducing catastrophe. We found that Klp5-Klp6 and Dis1 collaborate to reduce spindle elongation rates both before metaphase and during anaphase A to generate spindles of appropriate length. Additionally, we characterised *dis1*Δ cells at a permissive temperature for the first time. We discuss the mechanisms by which the kinesin-8 TBs control processivity, the role of MT control in spindle length control and the relative importance of Klp5-Klp6 and Dis1 in these processes.

## RESULTS

### A mutation in the ‘tailbox’ renders Klp5 temperature sensitive in *dis1*Δ cells

**Fig. 1.**
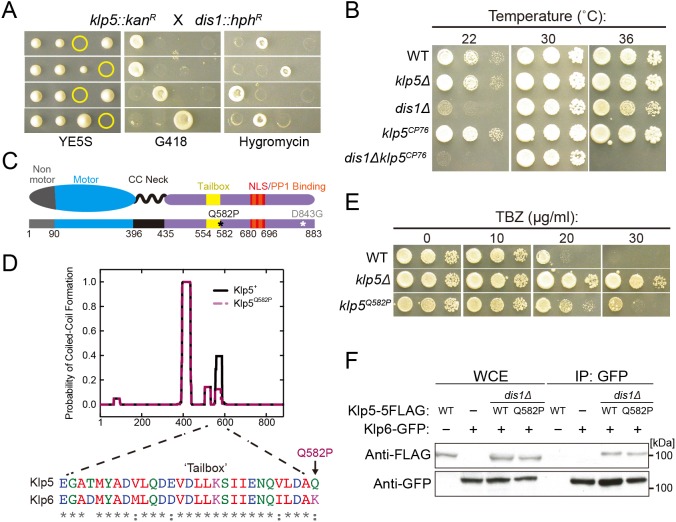
**Isolation and initial characterisation of a temperature-sensitive *klp5* TB mutant.** (A) Tetrad analysis of *klp5*Δ (*kan^R^*, G418 resistance) and *dis1*Δ (*hph^R^*, hygromycin resistance) after mating and sporulation, dissected on YE5S plates and incubated at 30°C for 3 days. Representative tetrads are shown and genotype was scored by replica plating to selective medium. Yellow circles indicate inviable meiotic products. (B) Serial dilution assay. YE5S plates were incubated at the indicated temperatures for 3 days. (C) Representation of Klp5 protein. Motor domain (blue) with N-terminal non-motor region (grey), CC neck linker (black) and the tail region (purple) containing the TB (yellow), nuclear localisation signal (NLS, red) and PP1-binding motifs (orange). Asterisks indicate locations of Q582P and D843G mutations. (D) Probability of CC formation for Klp5 protein sequence (*x*-axis gives amino acid position). Performed using COILS prediction software (Lupas et al., 1991) on MTIDK matrix using window 28 with a weight of 2.5 at heptad positions *a* and *d*. Solid black trace, WT Klp5 protein sequence; dashed purple trace, Klp5^Q582P^ protein sequence. Alignment of residues corresponding to the TB regions of Klp5 (amino acids 554-583) and Klp6 (amino acids 548-576) with amino acid similarity noted beneath each residue. (E) Serial dilution assay. YE5S plates were incubated at 36°C for 3 days. (F) Immunoprecipitation from cells expressing Klp5-FLAG (WT or Q582P), Klp6-GFP or both. Klp6-GFP was immunoprecipitated with the GFP-trap and immunoblotting was performed with anti-FLAG (top) or anti-GFP (bottom) antibodies against whole cell extracts (WCE) and Klp6-GFP immunoprecipitates.

We first verified the requirement of kinesin-8 and TOG proteins for viability by assessing the synthetic lethality between *klp5*Δ and *dis1*Δ and found that double mutants were inviable at a temperature permissive to cold-sensitive *dis1*Δ (Fig. 1A). Following this, we undertook a screen for temperature-sensitive mutants of *klp5* in the *dis1*Δ background. We obtained *klp5^CP76^*, a mutant that was not temperature-sensitive in the *dis1^+^* background nor rescued the *dis1*Δ cold-sensitive growth phenotype (Ohkura et al., 1988) (Fig. 1B).

We confirmed that lethality was not a result of instability of the mutant protein; Klp5^CP76^ levels were indistinguishable from those of wild-type (WT) protein in the *dis1*Δ background at restrictive temperature (Fig. S1A). Nucleotide sequencing of the *klp5^CP76^* allele revealed two missense mutations in the non-motor tail: Q582P at the end of the TB region (West et al., 2001) and D843G at the C-terminus (Fig. 1C). Through site-directed mutagenesis, we determined that Q582P was responsible for the temperature-sensitive phenotype of *klp5^CP76^* in *dis1*Δ cells (Fig. S1B). Thus, we pursued the *klp5^Q582P^* allele for subsequent analyses.

The kinesin-8 TB region is a stretch of 34 amino acids that is highly conserved between Klp5 and Klp6 (Fig. 1D, bottom) (West et al., 2001; Meadows et al., 2011) and is predicted to form a coiled coil (CC) based on sequence (Fig. 1D, top, black trace) (West et al., 2001). However, the physiological significance of the TB has not been addressed. Substituting proline for glutamine significantly reduced the probability of CC formation (Fig. 1D, top, purple trace) and conferred marked resistance to the MT-depolymerising drug thiabendazole (TBZ), characteristic of kinesin-8 malfunction (Fig. 1E) (West et al., 2001). It is formally possible that the TB, as a CC structure, participates in heterodimerisation of Klp5-Klp6; therefore, we assessed the interaction between Klp6 and Klp5^Q582P^ proteins and found that Klp6-GFP immunoprecipitated mutant and WT Klp5-5FLAG proteins equally well (Fig. 1F). Taken together, these data indicate that the TB mutation affects an essential kinesin-8 function in a manner distinct from Klp5-Klp6 complex formation.

### Klp5-Klp6 TBs are required for overall mitotic function

**Fig. 2.**
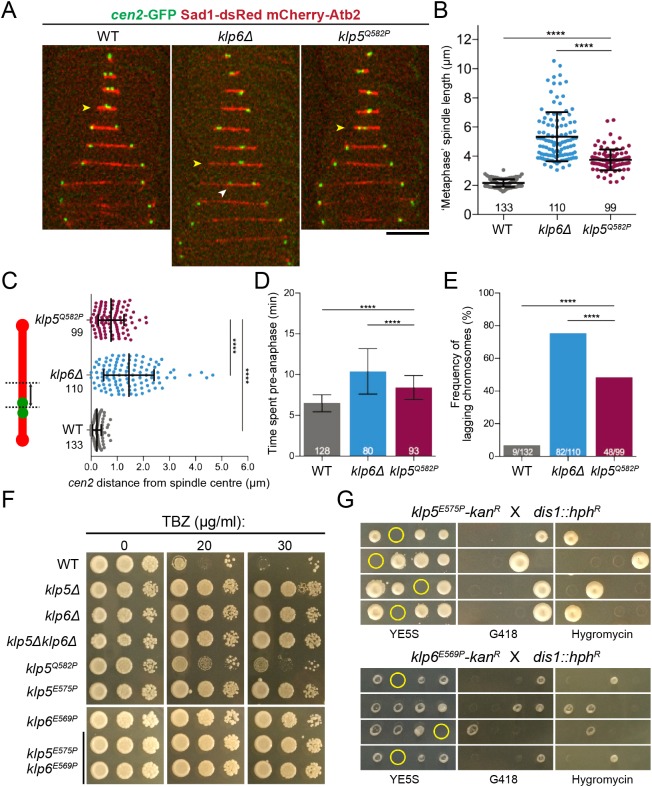
**The TB is required for overall kinesin-8 function.** (A) Representative kymographs of WT, *klp6*Δ and *klp5^Q582P^* cells expressing the centromere marker *cen2*-GFP (green) (Yamamoto and Hiraoka, 2003) along with an SPB marker Sad1-dsRed (red) (Hagan and Yanagida, 1995) and the tubulin marker mCherry-Atb2 (red) (Toda et al., 1984). Images were acquired at 2 min intervals at 36°C. Yellow arrowheads indicate metaphase; white arrowhead indicates lagging chromatid. Scale bar: 5 µm. (B) Metaphase spindle lengths of WT, *klp6*Δ and *klp5^Q582P^* cells. (C) Distribution of absolute distance of *cen2*-GFP signals from spindle centre (black arrow, left scheme) as a measure of congression profiles in WT, *klp6*Δ and *klp5^Q582P^* cells. (D) Time before the onset of anaphase A in WT, *klp6*Δ and *klp5^Q582P^* cells. (E) Frequency of lagging chromosomes during anaphase in WT, *klp6*Δ and *klp5^Q582P^* cells. The statistical significance of difference was determined using a Fisher's exact test. (F) Serial dilution assay. YE5S plates were incubated at 36°C for 3 days. (G) Tetrad analyses. YES5 plates were incubated at 30°C for 3 days. Representative tetrads are shown; genotype was scored by replica plating to selective medium. Yellow circles indicate inviable meiotic products. For B-E, the number of samples analysed is shown on the graphs; error bars indicate standard deviation; ****P<0.0001.

To investigate the role of the kinesin-8 TB, we observed mitosis in *klp5^Q582P^* cells by time-lapse microscopy at restrictive temperature (Fig. 2A). Metaphase spindle length was increased in *klp5^Q582P^* cells (3.75±0.71 µm versus 2.12±0.26 µm in WT cells) but less than in *klp6*Δ cells, which lacked mitotic kinesin-8 function (5.34±1.69 µm, Fig. 2B). Also, *klp5^Q582P^* negatively affected chromosome congression, with chromosomes displaced further from the spindle centre compared with WT (0.77±0.49 µm versus 0.22±0.16 µm), yet did not deviate as far as in *klp6*Δ cells (1.45±0.96 µm, Fig. 2C).

The onset of anaphase was delayed by *klp5^Q582P^*; cells spent an average of 8.43±1.74 min pre-anaphase, whereas WT cells spent 6.50±1.03 min and *klp6*Δ cells took an extended 10.40±2.78 min (Fig. 2D). Furthermore, the frequency of lagging chromatids was high in *klp5^Q582P^* cells (48.50%) but lower than in *klp6*Δ cells (75.45%) (Fig. 2E). Together, these results imply that *klp5^Q582P^* is a broad hypomorphic allele, perturbing but not abolishing all mitotic functions of kinesin-8.

To further validate the necessity of this CC motif, we sought to create null alleles by mutating central TB residues, generating *klp5^E575P^* and *klp6^E569P^* (Fig. 1D; Fig. S2A). These mutants phenocopied the TBZ-resistance of deletion cells, indicating severe loss of kinesin-8 function (Fig. 2F). Consistently, both *klp5^E575P^* and *klp6^E569P^* were nonconditionally lethal with *dis1*Δ (Fig. 2G). In line with previous reports (Gergely et al., 2016), all *klp5/6* mutants caused extreme nuclear envelope (NE) deformation around long spindles before anaphase onset (denoted by Cut11-GFP foci at SPBs) (West et al., 1998; Walde and King, 2014) (Fig. S2B, top). Pre-anaphase spindle length and duration were both increased in *klp5^E575P^* cells compared with *klp5^Q582P^*, yet were milder compared with *klp5/6*Δ cells (Fig. S2B, bottom). Together, these results confirm a vital role for the TBs of Klp5 and Klp6 in the mitotic function of kinesin-8. It should be noted that pre-anaphase spindle length and duration (Fig. S2B) in addition to severity of spindle protrusion length (Fig. S2C) were not significantly different between *klp5*Δ and *klp6*Δ cells, consistent with their function as a heterodimer (Garcia et al., 2002b; Unsworth et al., 2008).

### Klp5-Klp6 increases catastrophe frequency, and kinesin processivity depends on the TB motif

**Fig. 3.**
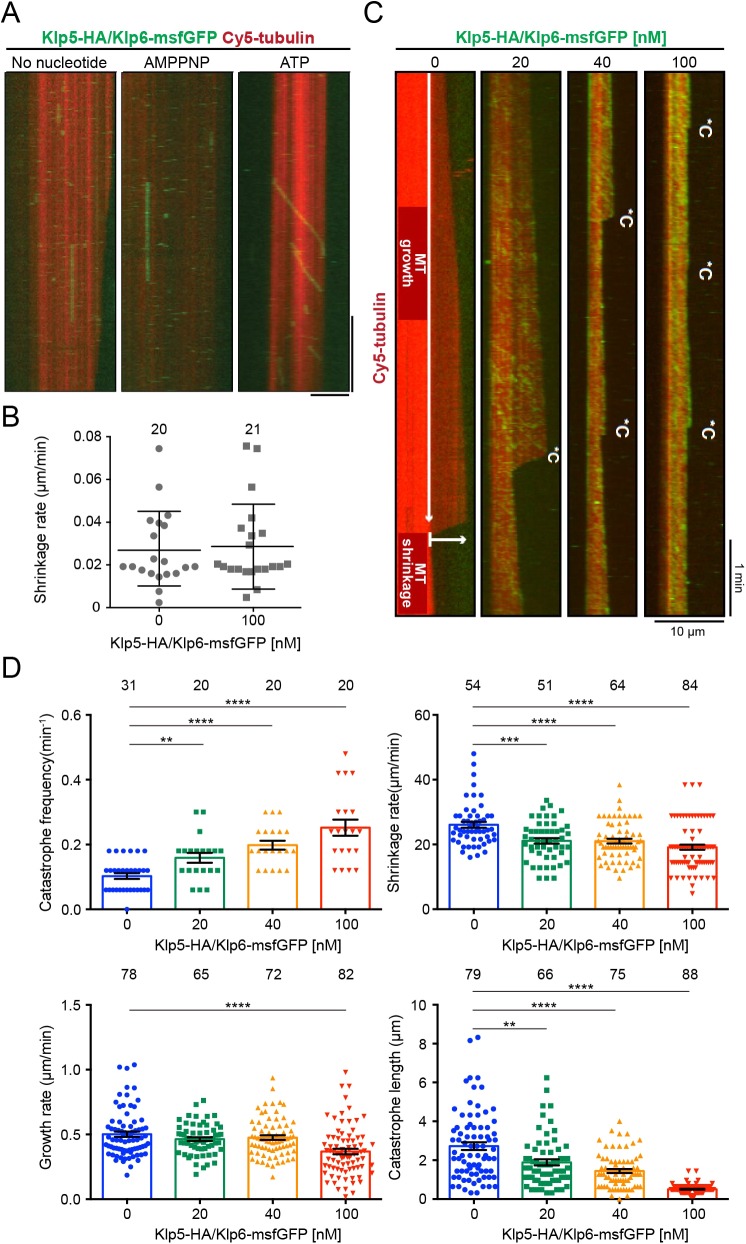
**Kinesin-8 increases catastrophe frequency and relies on the TB motif for processive plus-end-directed motility *in vitro*.** (A) Representative dual-colour TIRF-M kymographs depicting Cy5-labelled, GMPCPP-stabilised MTs (red) and 5 nM Klp5-HA/Klp6-msfGFP (green) in the presence of no nucleotide (left), 2 mM of the non-hydrolysable ATP analogue AMPPNP (middle) or 1 mM ATP (right). Scale bars: vertical, 1 min; horizontal, 10 µm. (B) Mean shrinkage rate of GMPCPP-stabilised MTs in the absence or presence of 100 nM Klp5-HA/Klp6-msfGFP. Data points represent shrinkage events; mean±s.d. are indicated. (C) Representative dual-colour TIRF-M kymographs depicting growth of Cy5-labelled MTs in the presence of 7.5 µM Cy5-labelled tubulin and 1 mM ATP plus 0, 20, 40 or 100 nM Klp5-HA/Klp6-msfGFP. *C denotes catastrophe events, where the polymerising MT (darker red) shrinks back to the seed (brighter red). MT growth occurs from the seed in the *y*-direction over time (MT growth). MT shrinkage indicates that an MT shrinks in length in the *x*-direction after a catastrophe event. (D) MT dynamics parameters in the presence of Klp5-HA/Klp6-msfGFP. Top left, mean catastrophe frequency calculated from individual MTs undergoing periods of growth. Sample sizes denote number of MTs assessed. Top right, mean MT shrinkage rate. Bottom left, mean MT growth rate. Bottom right, mean catastrophe length. For both shrinkage rate and growth rate analyses, sample numbers denote total number of events measured. Mean±s.e.m. values are indicated. The statistical significance of difference was determined using a Student's unpaired *t*-test; **P<0.01; ***P<0.001; ****P<0.0001.

Although earlier studies confirmed fission yeast kinesin-8 to be a plus-end-directed motor complex (Grissom et al., 2009; Erent et al., 2012), its activity on dynamically growing MTs has not been investigated. We bacterially co-expressed and purified a recombinant, full-length Klp5-HA/Klp6-msfGFP complex (Fig. S3A, see Materials and Methods). To verify its motor activity, we used total internal reflection fluorescence microscopy (TIRF-M) to visualise the kinesin on MTs stabilised with the hydrolysable GTP analogue GMPCPP in the absence or presence of ATP or the non-hydrolysable analogue AMPPNP. The complex moved in a unidirectional manner only in the presence of ATP (Fig. 3A). In accordance with previous observations (Grissom et al., 2009; Erent et al., 2012), the complex did not enhance the shrinkage rate of GMPCPP-stabilised MTs (Fig. 3B). We then assessed the motility of the Klp5-HA/Klp6-msfGFP complex on dynamic MTs by TIRF-M. MTs were grown from immobilised and GMPCPP-stabilised MT seeds in the presence of Cy5-labelled tubulin and GTP (Bieling et al., 2010) (see Materials and Methods). We found that the kinesin displayed a processive motility towards the clearly growing MT plus end (Fig. S3B).

Next, we assessed the effects of the Klp5-HA/Klp6-msfGFP complex on the behaviour of individual MTs by TIRF-M and quantified the dynamic instability parameters at varying concentrations of kinesin-8 (Fig. 3C). The frequency of MT catastrophe (switch from growth to shrinkage) increased in a dose-dependent manner, with observable effects even at a low concentration (20 nM) of Klp5-HA/Klp6-msfGFP (Fig. 3C,D, top left). In contrast, MT shrinkage rate decreased slightly but significantly, whereas growth rate remained similar at lower concentrations and decreased only at 100 nM (Fig. 3D, top right and bottom left, respectively). It is also noteworthy that catastrophe length significantly decreased in a dose-dependent manner (Fig. 3D, bottom right). Quantitative values of MT dynamics are summarised and shown in Table 1. These results are in line with those recently reported for *Drosophila* Klp67A (Edzuka and Goshima, 2019) and suggest that the major mechanism of MT destabilisation by Klp5-Klp6 is through the induction of catastrophe events.

**Table 1.**
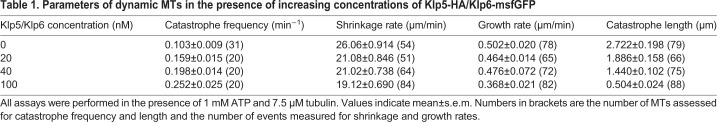
**Parameters of dynamic MTs in the presence of increasing concentrations of Klp5-HA/Klp6-msfGFP**

**Fig. 4.**
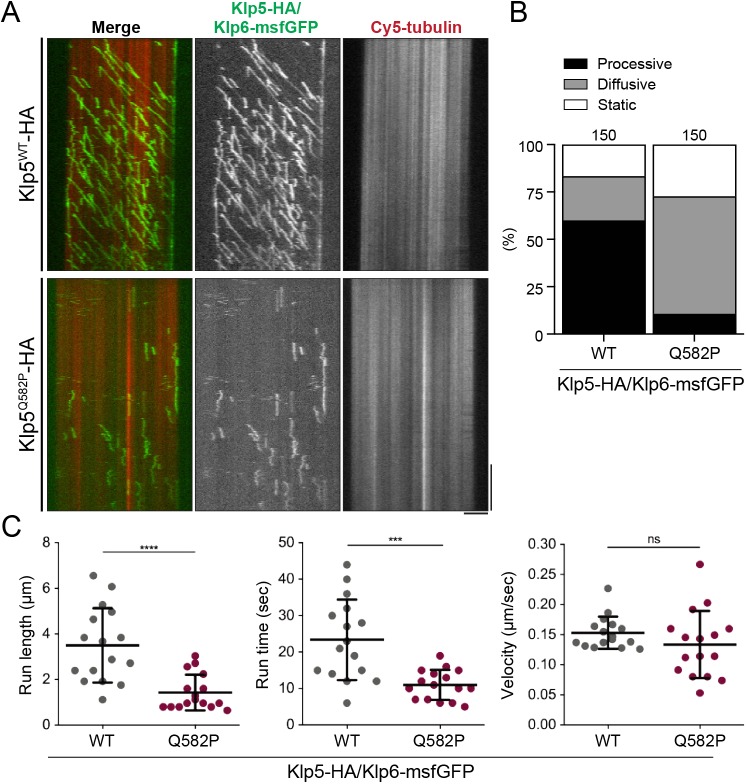
**The kinesin-8 TB is required for processivity *in vitro*.** (A) Representative dual-colour TIRF-M kymographs depicting Cy5-labelled, taxol-stabilised MTs (red) and 10 nM Klp5-HA/Klp6-msfGFP (green), either WT or Q582P mutant in the presence of 1 mM ATP. Scale bars: vertical, 1 min; horizontal, 5 µm. (B) Categories of fluorescently tagged kinesin-8 binding events. Percentage frequencies of processive (black), diffusive (grey) or static (white) kinesin-8 runs by the WT or Q582P mutant complex are given; 150 samples of each were analysed. (C) Kinesin-8 run length, run time and calculated velocity of processive events (*n*=16). Mean±s.d. values are indicated. ***P=0.0004, ****P<0.0001; ns, *P*=0.1801.

With the functionality of our Klp5-Klp6 construct validated, we co-expressed and purified a recombinant Klp5^Q582P^-HA/Klp6-msfGFP heterodimer alongside the WT construct (Fig. S4A). Gel filtration elution profiles of the WT and mutant complexes were very similar, indicating that the Klp5^Q582P^-HA/Klp6-msfGFP complex is not aggregated nor misfolded (Fig. S4B,C). TIRF-M observation of this mutant on taxol-stabilised MTs revealed striking differences in the behaviour of WT and Q582P complexes (Fig. 4A). The WT complex showed frequent binding events and the subsequent processive runs exhibited clear directionality towards one end of the MT, identifying it as the plus end (Fig. 4A, top). In contrast, markedly fewer binding events were observed for the TB mutant complex and runs appeared less processive by visual inspection, exhibiting weaker directionality (Fig. 4A, bottom). We quantified the difference between the two kinesin-8 constructs on taxol-stabilised MTs by run behaviour, categorising runs as processive (smooth and continuous movement to one MT end), diffusive (back and forth movement with overall plus-end directionality) or static (Fig. S4D). The WT complex was largely processive (60% of runs) (Fig. 4B). In contrast, the TB mutant complex was mostly diffusive (62% of runs) and both complexes displayed static binding events, the frequency of which was slightly elevated by the TB mutation (Fig. 4B). This reduction of processivity and increase in diffusive movement on taxol-stabilised MTs was also mirrored by the TB mutant complex on dynamic MTs (Fig. S4E).

We then measured the run length, run time and velocity from kymographs of processive runs to check for any inherent differences in this behaviour. Interestingly, this analysis revealed that run time and length were markedly reduced (∼50%) by the TB mutation (Fig. 4C, left and centre). However, the velocity of these runs was in fact similar to that of the WT complex (0.153±0.027 µm/s for WT versus 0.134±0.056 µm/s for Q582P) (Fig. 4C, right). These data show that the TB region of Klp5 plays a crucial role in the processivity of fission yeast kinesin-8, but not through increasing velocity.

### Klp5-Klp6 and Dis1 cooperate to limit metaphase spindle length by restraining pre-anaphase spindle elongation rates

**Fig. 5.**
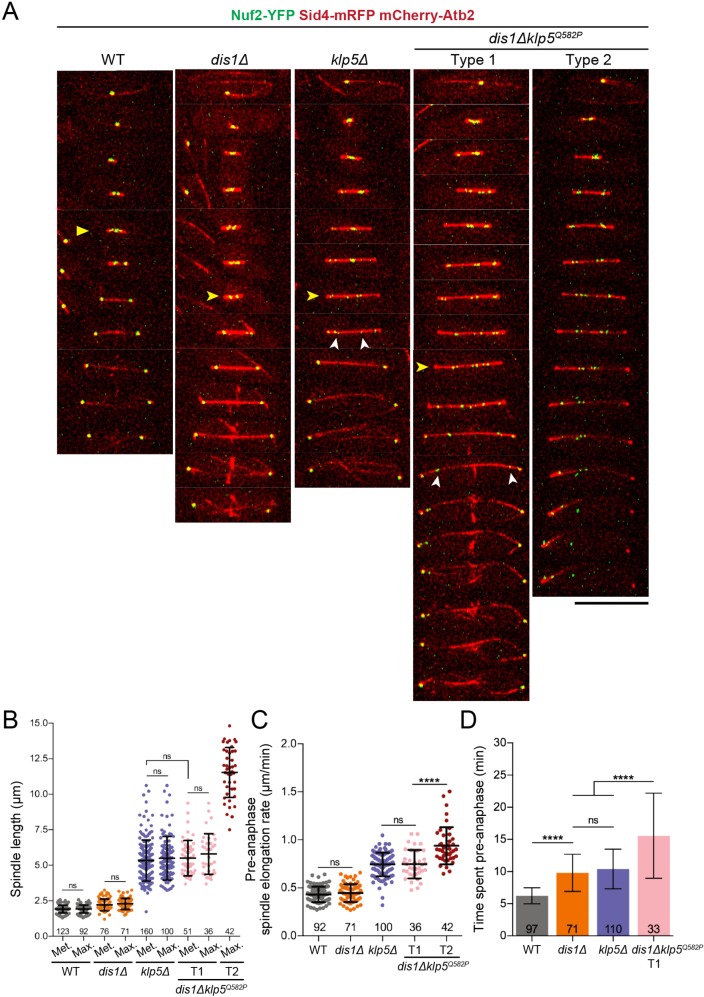
**Kinesin-8 and Dis1 control metaphase spindle length by limiting pre-anaphase spindle elongation for timely transition into anaphase.** (A) Representative kymographs of WT, *dis1*Δ, *klp5*Δ and *dis1*Δ*klp5^Q582P^* cells expressing the KT marker Nuf2-YFP (green) (Nabetani et al., 2001) along with an SPB marker Sid4-mRFP (red) and the tubulin marker mCherry-Atb2 (red). Images were acquired at 2 min intervals at 36°C. Yellow arrowheads, metaphase; white arrowheads, lagging chromatids. Scale bar: 10 µm. (B) Metaphase (Met) and maximum (Max) pre-anaphase spindle lengths of WT, *dis1*Δ, *klp5*Δ and *dis1*Δ*klp5^Q582P^* cells. For Met *klp5*Δ versus Met type 1 *dis1*Δ*klp5^Q582P^*, difference was not significant (ns), *P*=0.3138. For Met versus Max comparison within cell types, *P*-values were 0.9229, 0.1910, 0.4702 and 0.3161 from left to right on graph, all ns. (C) For maximum pre-anaphase spindle elongation rates in WT versus *dis1*Δ and *klp5*Δ versus type 1 *dis1*Δ*klp5^Q582P^* cells, *P*-values were *P*=0.4176 and 0.7338, respectively; ****P<0.0001. (D) Time before the onset of anaphase A in WT, *dis1*Δ, *klp5*Δ and type 1 *dis1*Δ*klp5^Q582P^* cells. ****P<0.0001; ns, *P*=0.1610. For B-D, the number of samples analysed is shown; mean±s.d. values are indicated.

Using our conditional *klp5^Q582P^* allele, we next investigated the cause for synthetic lethality between Klp5-Klp6 and Dis1 in detail by time-lapse imaging of double mutants at restrictive temperature (Fig. 5A). Similar to *klp5*Δ, double mutants showed a spread of chromosomes over long spindles and spindle bending. Closer inspection revealed two populations of *dis1*Δ*klp5^Q582P^* cells: 37.1% (64/105) underwent anaphase, albeit after a long delay, whereas 62.9% (105/169) continued spindle elongation without chromosome segregation. For clarity, we split these double mutant cells into two populations, type 1 and type 2, where the former could complete anaphase and the latter retained non-disjoined sister chromatids. Eventually, these chromatids moved together towards one SPB in type 2 cells (Fig. 5A).

Analysis of spindle lengths changes exposed further differences between the *dis1*Δ*klp5^Q582P^* cell types. Type 1 cells underwent prolonged spindle elongation before the distinctive rapid growth during anaphase B, whereas type 2 cells sustained spindle elongation for much longer. This was followed by reductions in spindle length as a result of spindle breakage (Fig. S5). In contrast, *dis1*Δ cells maintained short spindles for longer before anaphase onset at restrictive temperature (Fig. 5A; Fig. S5). Our data therefore indicate that kinesin-8 and Dis1 work together to coordinate proper spindle length changes during mitosis.

Upon closer inspection, *dis1*Δ had little effect on metaphase spindle length (2.12±0.41 versus 1.92±0.27 µm for WT) but type 1 *dis1*Δ*klp5^Q582P^* metaphase spindles lengths approached those of *klp5*Δ cells (5.51±1.24 and 5.34±1.44 µm, respectively) (Fig. 5B). We postulated that type 2 spindles were too long for chromosome biorientation and segregation to proceed, implying the existence of an upper limit for metaphase spindle length, which can be achieved in type 1 cells. Indeed, in all cases, the maximum spindle lengths reached during pre-anaphase were no different to metaphase lengths, but type 2 spindles were nearly twice as long (Fig. 5B; no metaphase spindle length measurements for type 2 cells could be taken as there was no metaphase-anaphase transition). The fact that *dis1*Δ is additive for spindle length in type 2 but not type 1 cells suggests that differences in *klp5^Q582P^* functionality are responsible for the distinction between metaphase spindles.

To understand how these MAPs regulate metaphase spindle length, we compared maximum pre-anaphase spindle elongation rates. Fission yeast pre-anaphase is classically divided into two phases: increasing spindle length (0 to ∼2.5 µm, phase 1) and constant spindle length (∼2.5 to ∼3 µm, phase 2) (Nabeshima et al., 1998). However, we could not accurately assign these two phases based on spindle length in mutant cells and, as such, assessed spindle behaviour ‘pre-anaphase’ instead. Spindles in type 2 cells elongated the quickest at 0.94±0.19 µm/m whereas rates were the same for *klp5*Δ and type 1 cells (0.75±0.12 and 0.75±0.15 µm/min, respectively) (Fig. 5C). Elongation rates were also similar for WT and *dis1*Δ cells (0.43±0.08 and 0.45±0.09 µm/min, respectively). These data suggest that kinesin-8 and Dis1 enforce an upper length limit on the metaphase spindle by restraining pre-anaphase elongation so that the spindle is competent for accurate chromosome segregation. It is noteworthy that the role of Dis1 in restraining spindle length becomes apparent only when kinesin-8 function is abrogated.

Additionally, the absence of both kinesin-8 and Dis1 had deleterious effects on the timing of the metaphase–anaphase transition in an additive manner: type 1 cells took an average of 15.58±6.61 min to transition, whereas *dis1*Δ and *klp5*Δ cells took 9.80±2.89 and 10.42±3.09 min, respectively (Fig. 5D). This result suggests that kinesin-8 and Dis1 make collaborative, distinct contributions to the generation of satisfactory KT-MT attachments to initiate punctual anaphase onset.

### Klp5-Klp6 and Dis1 suppress spindle elongation in anaphase A to prevent lagging chromosomes

**Fig. 6.**
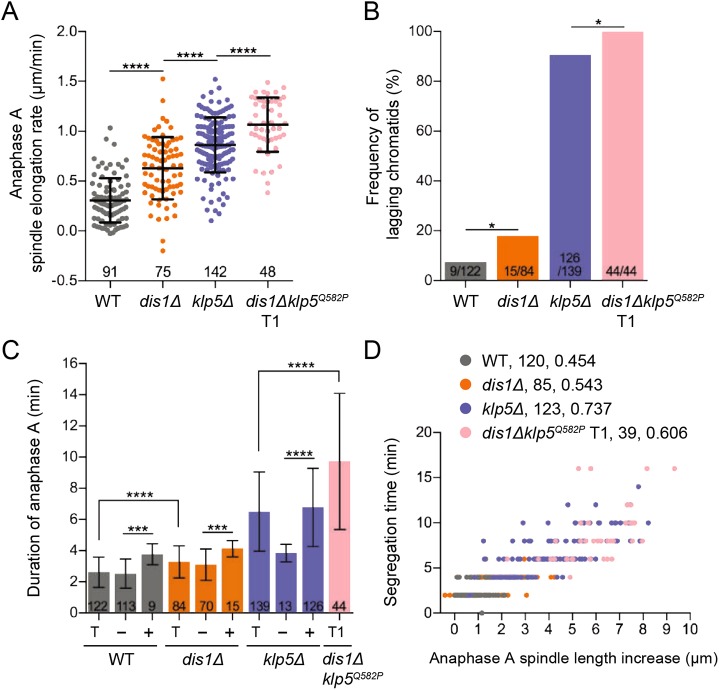
**Kinesin-8 and Dis1 limit spindle elongation during anaphase A to ensure coordinated chromosome segregation.** (A) Maximum spindle elongation rates during anaphase A in WT, *dis1*Δ, *klp5*Δ and type 1 *dis1*Δ*klp5^Q582P^* cells. ****P<0.0001. (B) Frequency of lagging chromatids during anaphase A in WT, *dis1*Δ, *klp5*Δ and type 1 *dis1*Δ*klp5^Q582P^* cells. The statistical significance of difference was determined using a Fisher's exact test; *P=0.0270 and 0.0402 from left to right. (C) Duration of anaphase A was calculated for the total number of cells (T) and cells without (−) or with (+) at least one lagging chromatid in WT, *dis1*Δ, *klp5*Δ and type 1 *dis1*Δ*klp5^Q582P^* (T1) cells. ***P=0.0003 and 0.0004 from left to right; ****P<0.0001. For A-C, the number of samples analysed is shown; error bars indicate standard deviation. (D) Duration of anaphase A was plotted against the corresponding increase in spindle length for WT, *klp5*Δ and type 1 *dis1*Δ*klp5^Q582P^* cells. Genotypes are followed by the number of XY pairs plotted and the corresponding Spearman correlation for each.

Next, we assessed any perturbations to anaphase resulting from the absence of kinesin-8 and Dis1. In contrast to the normally limited spindle elongation during anaphase A, where the maximum rate in WT cells was 0.31±0.22 µm/min, *dis1*Δ doubled the rate to 0.63±0.31 µm/min and the absence of kinesin-8 function almost tripled the rate to 0.86±0.28 µm/min (Fig. 6A). These effects were additive in *dis1*Δ*klp5^Q582P^* cells, where anaphase A spindle elongation reached 1.07±0.27 µm/min. This result again implies novel, distinct roles for Dis1 and Klp5-Klp6 in restraining spindle growth during anaphase A, challenging a simple idea of spindle length control by antagonistic MT polymerisation and depolymerisation by Dis1 and kinesin-8, respectively.

Remarkably, the frequency of lagging chromatids increased from 7.38% in WT to 17.86% in *dis1*Δ cells and rose from 90.65% to 100% when Dis1 was removed from cells lacking functional Klp5-Klp6 (Fig. 6B), implicating Dis1 alongside kinesin-8 in orchestrating coordinated chromosome segregation. Furthermore, the duration of anaphase A increased from 2.62±0.96 min in WT cells to 3.27±1.01 min in the absence of Dis1, and to 6.50±2.54 min in *klp5*Δ cells (Fig. 6C). Again, double mutant cells had the most severe extension of segregation time, taking 9.73±4.37 min. We found that the duration of anaphase A was extended by the presence of a lagging chromatid in all strains, where *dis1*Δ*klp5^Q582P^* cells took the longest to complete segregation as all cells exhibited lagging chromatids (Fig. 6C). Subsequently, we found that the degree of increase in spindle length during anaphase A correlated positively with chromosome segregation time in all cell types, implying that aberrant spindle elongation prevents timely segregation of sister chromatids, generating lagging chromatids (Fig. 6D).

### Elongated spindles remain coupled to mitotic events in single- and double-mutant cells

**Fig. 7.**
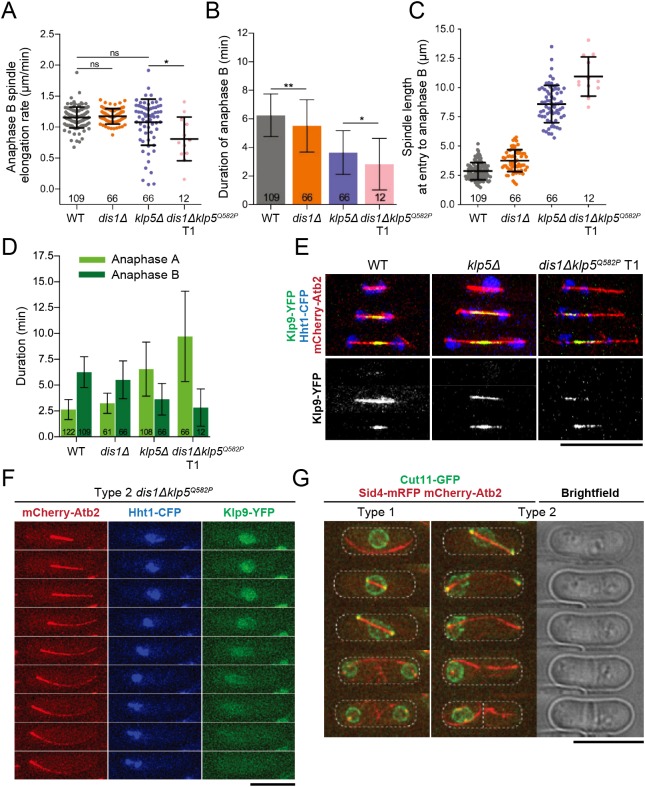
**Anaphase B is shortened to compensate for anaphase A in kinesin-8 single and double mutants but remains coupled to mitotic events.** (A) Maximum spindle elongation rates during anaphase B in WT, *dis1*Δ, *klp5*Δ and type 1 *dis1*Δ*klp5^Q582P^* cells. *P=0.0160. (B) Duration of anaphase B was calculated for WT, *dis1*Δ, *klp5*Δ and type 1 *dis1*Δ*klp5^Q582P^* cells. *P=0.0362, **P=0.0070. (C) Spindle length at anaphase B onset in WT, *dis1*Δ, *klp5*Δ and type 1 *dis1*Δ*klp5^Q582P^* cells. (D) Duration of anaphase A and B was compared for WT, *dis1*Δ, *klp5*Δ and type 1 *dis1*Δ*klp5^Q582P^* cells. For A-D, the number of samples analysed is shown; mean±s.d. values are indicated. (E,F) Representative kymographic images of Klp9-YFP (green) recruitment to spindles in cells also expressing the histone marker Hht1-CFP (blue) and the tubulin marker mCherry-Atb2 (red). Images were acquired at 2 min intervals at 36°C. (E) Klp9-YFP recruitment events in WT, *klp5*Δ and type 1 *dis1*Δ*klp5^Q582P^* cells, where the second frame depicts the first instance of midzone localisation, with one frame before and one frame after. (F) Image sequence shows a representative type 2 *dis1*Δ*klp5^Q582P^* cell undergoing mitosis, where frames are 4 min apart. (G) *dis1*Δ*klp5^Q582P^* cells expressing Cut11-GFP along with Sid4-mRFP and mCherry-Atb2 were imaged every 2 min at 36°C; images shown are at 16 min intervals. Scale bars: 10 µm.

We also checked for perturbations to anaphase B in our mutants as Klp5-Klp6 becomes restricted to the spindle midzone and thus might have a role at this point in mitosis (Garcia et al., 2002b; West et al., 2002; Lucena et al., 2015). Remarkably, single deletion mutants exhibited normal anaphase B spindle elongation; rates were similar in WT, *dis1*Δ and *klp5*Δ cells (1.16±0.17, 1.18±0.12 and 1.08±0.37 µm/min, respectively) (Fig. 7A). The rate was only significantly slower in double mutants at an average of 0.81±0.35 µm/min (Fig. 7A). We also found that the duration of anaphase B was shortest in *dis1*Δklp5^Q582P^ cells (Fig. 7B). Although these data suggest that anaphase B is perturbed in double mutant cells, these spindles were found to be longer initially because spindle elongation occurred earlier in mitosis (Fig. 7C). We then postulated that cells that spent longer in anaphase A tended to spend less time in anaphase B: comparing the duration of these stages revealed this to be the case (Fig. 7D). Rather than Dis1 and Klp5-Klp6 directly contributing to spindle elongation in anaphase B, we propose that precocious spindle elongation during anaphase A diminishes the need for further elongation. This notion is supported by the fact that there were fewer *dis1*Δklp5^Q582P^ cells able to perform anaphase B for analysis, as indicated by the smaller sample sizes (Fig. 7A-D).

Observed spindle elongation during chromosome segregation in Klp5-Klp6 mutants has previously led to the hypothesis that anaphase B is initiated before anaphase A in these cells (West et al., 2002). Certainly, in kinesin-8 mutant cells spindles approach cell length prior to the first signs of chromosome segregation (Fig. 5A; Fig. S5), a phenomenon normally limited to anaphase B. To delineate whether anaphase B spindle elongation is initiated precociously, we visualised Klp9, the plus-end-directed kinesin-6 that accumulates at the spindle midzone to drive outward spindle sliding during anaphase B (Fu et al., 2009; Meadows et al., 2017; Yukawa et al., 2019), at restrictive temperature in our kinesin-8 mutants. Similar to WT and *klp5*Δ, type 1 cells recruited Klp9-YFP to the midzone only following the onset of chromosome segregation and never before (Fig. 7E), confirming that anaphase A still precedes anaphase B in mutant cells. Remarkably, Klp9-YFP could not be detected on type 2 spindles; only pre-anaphase nucleoplasmic signals were observed (Fig. 7F). Consistent with this result, in type 2 cells, signals of Cdc13-sfGFPcp (cyclin B with a circularly permutated superfolder GFP) (Kamenz et al., 2015), which normally disappear upon anaphase onset, persisted within the nucleus and on spindle microtubules (44 out of 51 type 2 cells retained robust Cdc13 signals; Fig. S6A). Therefore, we conclude that the long spindle phenotypes observed in single or double kinesin-8 mutants are not a result of the premature onset of anaphase B driven by Klp9 and are still pre-anaphase, as indicated by the presence of Cdc13-sfGFPcp. Thus, spindle behaviour is still coupled with chromosome events in that anaphase B does not occur in the absence of chromosome segregation. Furthermore, extremely long pre-anaphase type 2 spindles are a direct result of the absence of Klp5-Klp6 and Dis1 function.

### Chromatids remain non-disjoined because of displacement by the rapidly elongating spindle and cells ultimately become cut

To conclude, we observed the terminal phenotype in *dis1*Δ*klp5^Q582P^* cells. It was found that long spindles in double mutant cells caused deformation of the NE (Fig. 7G). Interestingly, type 2 cells displayed displacement of the undivided nucleus to one side of the cell, perhaps through pushing force generated by elongating spindles in contact with the cell cortex (Syrovatkina and Tran, 2015).

We also checked whether nuclear displacement was derived from inherent differences between mitotic SPBs (e.g. age, history). However, there was no bias in which SPB non-disjoined chromatids associated with, as judged by the localisation of Pmo25-GFP (Kanai et al., 2005), a marker for asymmetrical activity of the septation initiation network (SIN) on the daughter SPB (Krapp and Simanis, 2008) (Fig. S6B). Therefore, non-disjoined chromosomes did not show a biased association with either mother or daughter SPBs, indicating that the asymmetry of the terminal phenotype arises from a different source within the spindle. We note that our data infer that the SIN is active in type 2 cells in the absence of chromosome segregation, implying that these cells exhibit the ‘cut’ phenotype (Yanagida, 1998).

## DISCUSSION

Our study provides mechanistic insight into Klp5-Klp6 function and its collaboration with Dis1 in spindle length control. We found that the non-motor Klp5-Klp6 TB promotes the processivity required for general function. We demonstrated that Klp5-Klp6 induces MT catastrophe and not shrinkage to regulate MT length. We then identified a synergistic relationship between kinesin-8 and Dis1 by which they impose an upper limit on metaphase spindle length through restraining pre-anaphase spindle elongation. We also discovered hitherto unknown roles for Klp5-Klp6 and Dis1 in maintaining spindle length during anaphase A to prevent the appearance of lagging chromosomes. Overall, we demonstrate the importance of spindle length control in chromosome segregation and viable passage through mitosis.

### The kinesin-8 TB contributes to processivity

Our mutagenesis experiments found the Klp5-Klp6 TB region important for overall mitotic function and our *in vitro* reconstitution demonstrated this is executed through increasing run time and length for motor processivity. The decrease in mutant complex processivity *in vitro* accounts for the general hypomorphic phenotype of *klp5^Q582P^* cells; fewer complexes are able to reach MT plus ends, reducing the amount of kinesin-8 to a suboptimal level for control of MT length. We envisage that *dis1*Δ*klp5^Q582P^* cells present two distinct phenotypes as a result of variations in the amount of Klp5-Klp6 at MT plus ends. The local concentration is higher in type 1 cells than that in type 2 cells, leading to extremely long spindles and chromosome nondisjunction in the latter.

Like other family members, Klp5-Klp6 might contain secondary MT-binding sites in the tail. Kif18A uses this to strengthen MT lattice interaction for increased processivity (Mayr et al., 2011; Stumpff et al., 2011), whereas Kif18B relies on the site for plus-tip targeting (McHugh et al., 2018). The Kip3 tail improves run lengths and retention on MT plus ends but does not increase velocity (Su et al., 2011), similar to the results we present here. Even so, it is unlikely that the TB is directly involved in secondary MT binding because of its predicted CC structure, yet it is possible that TB disruption could alter tail structure, potentially indirectly affecting such a secondary interaction site.

The Klp5^Q582P^ complex could exhibit reduced processivity as a result of strengthened auto-inhibition. The binding of human kinesin-1 to MTs undergoes tail-motor auto-inhibition that is released by tail–cargo interactions (Friedman and Vale, 1999). Further biochemical study is required to accurately dissect how exactly the TB confers processivity to Klp5-Klp6.

### Fission yeast kinesin-8 induces catastrophe to destabilise MTs

Using a highly pure full-length recombinant Klp5-Klp6 complex, we provide the first direct evidence that kinesin-8 increases catastrophe frequency of dynamic MTs, where an increase in frequency is accompanied by a reduction in the length of growing MTs undergoing catastrophe. Higher resolution imaging is required to categorically confirm whether Klp5-Klp6 reduces catastrophe length by dampening plus-end MT dynamics, as suggested for human Kif18A (Du et al., 2010; Stumpff et al., 2011). Our data is in line with previous observations that *klp5*Δ/*klp6*Δ reduced the catastrophe frequency of cytoplasmic MTs (Unsworth et al., 2008; Tischer et al., 2009; Erent et al., 2012; Meadows et al., 2018; Niu et al., 2019).

Similarly, Kif18B induces catastrophe of MTs to control astral MT length for spindle centring (McHugh et al., 2018). In contrast, how Kif18A influences MT lengths is under debate: some studies report direct MT depolymerisation (Mayr et al., 2011; Locke et al., 2017), whereas others show dampening of MT dynamics (Du et al., 2010; Stumpff et al., 2011). Kip3 clearly depolymerises MTs, but whether tubulin removal depends on motility is in question (Varga et al., 2006; Gupta et al., 2006; Arellano-Santoyo et al., 2017). We have demonstrated that Klp5-Klp6 regulates MT length by inducing catastrophes and not by increasing shrinkage rate, but the underlying mechanism remains to be elucidated. Our results build on the growing body of evidence that kinesin-8 proteins are a structurally and functionally diverse family of motors.

### Klp5-Klp6 collaborate with Dis1 to establish a metaphase spindle capable of chromosome segregation

We found that Klp5-Klp6 reduces spindle elongation rate from the onset of mitosis to generate short metaphase spindles for proper chromosome segregation and that Dis1 supports this. This result is surprising in that Dis1 is classically described as an MT polymerase (Matsuo et al., 2016) but we found that *dis1*Δ cells behaved similarly to WT cells pre-anaphase. However, we demonstrate that Dis1 limits early spindle elongation to establish a viable metaphase spindle in the absence of kinesin-8 function. This is in stark contrast to the logical antagonistic relationship expected from MAPs with opposing MT activities. We recently found that, like other TOG proteins, Dis1 catalyses MT shrinkage *in vitro* in the absence of free tubulin (Matsuo et al., 2016); loss of this activity would increase spindle length *in vivo*. In kinesin-8 mutants, spindle MTs are stabilised and longer, which could limit the availability of free tubulins. Intriguingly, Dis1 appears to promote MT shortening for KT retrieval during the first meiotic division; however, this is likely to be mediated through the Dam1 kinetochore/MAP complex (Kakui et al., 2013). Therefore, it seems likely that Dis1 acts in a dual manner, promoting MT polymerisation or depolymerisation depending on cellular context, whereas Klp5-Klp6 appears to be the major negative regulator of pre-anaphase spindle length. It should also be noted that the absence of Klp5-Klp6 and Dis1 from crowded MT plus ends could allow increased recruitment of the Alp14-Alp7 MT polymerase complex, leading to increased MT polymerisation.

Both Dis1 and Klp5-Klp6 might also limit outward spindle growth by maintaining proper KT-MT attachments through their interaction with Ndc80 (Hsu and Toda, 2011; Tang and Toda, 2015), generating inward spindle force. Indeed, loss of this inward force through mutations in KT components produces longer spindles (Goshima et al., 1999). These ideas raise a general question regarding the relative contributions of direct active MT length control and passive inward force generation through proper KT-MT attachments to spindle length control that requires further dissection.

### Anaphase A spindle control prevents the appearance of lagging chromatids

Our conditional mutant also allowed us to study the role of Klp5-Klp6 and Dis1 during anaphase in type 1 cells. Aberrant spindle elongation during anaphase A has previously been described for *klp5*Δ*/klp6*Δ cells (West et al., 2002). We verified this phenomenon and report it, less severely, in *dis1*Δ cells. Furthermore, these phenotypes were additive, indicating that Klp5-Klp6 and Dis1 maintain the anaphase A spindle in distinct ways. During anaphase A, tension is lost at KTs and KT-MTs are actively shortening; therefore, KT-MTs are unlikely to be a site of spindle length control at this point (Grishchuk and McIntosh, 2006). Thus, Klp5-Klp6 and Dis1 might destabilise MT plus ends in the iMT overlap/midzone to control spindle length during anaphase A. In support of this proposition, Dis1 relocalises from KTs to the spindle during anaphase (Nabeshima et al., 1995; Decottignies et al., 2001), which could be accompanied by a switch to a more MT destabilising role. We found that Klp5-Klp6 and Dis1 control anaphase A spindle length to prevent lagging chromosomes; this is achieved by ensuring SPBs do not move further apart faster than KT-MTs pull chromosomes polewards.

In conclusion, our data highlights the importance of spindle length control, safeguarded by kinesin-8 and TOG proteins, not only in early mitosis for the stable biorientation of sister chromatids but also for continued length control into anaphase to ensure a coordinated and high-fidelity segregation of genetic material.

## MATERIALS AND METHODS

### Strains, media and genetic methods

Fission yeast strains used in this study are listed in Table S1. Cells were grown under standard conditions as previously described (Moreno et al., 1991). For all experiments, rich YE5S plates and media were used. Wild-type strain (513) was provided by P. Nurse (The Francis Crick Institute, London, UK). Tetrad dissection was performed on YE5S plates using the Singer MSM 400. Plates were incubated for 4 days before replica-plating onto appropriate plates for selection and genotyping. Serial dilution assays were performed using mid-log phase cells at a concentration of 2×10^6^ cells/ml, which were then serially diluted tenfold before spotting onto the appropriate YE5S plates. Plates were incubated for 3 days at the indicated temperatures.

### Production of *klp5* mutants in the *dis1*Δ background

A *klp5^+^-5FLAG-kanMX6* DNA fragment was amplified from purified genomic DNA extracted from the C-terminal-tagged strain. Error-prone PCR, using Vent DNA polymerase (New England Biolabs, Ipswich, MA, USA) supplemented with 10× deoxyguanosine triphosphate, was used to randomly mutagenise the *klp5^+^-5FLAG-kanMX6* fragment. Mutant fragments were then transformed into *dis1*Δ cells, where homologous recombination allowed replacement of the endogenous *klp5^+^* by the mutant fragment. Transformants were selected by plating cells onto YE5S with G418 (Geneticin; Sigma-Aldrich, St. Louis, MO, USA) and temperature sensitivity assessed by replica-plating to 36°C. Approximately ∼3500 colonies were screened for temperature sensitivity at 36°C and viability at 30°C.

### Fluorescence microscopy and sample preparation

Fluorescence microscopy images were acquired using one of two DeltaVision wide-field inverted epifluorescence systems (GE Healthcare, Chicago, IL, USA). Live cells were imaged using an Olympus IX71 microscope comprised of an Olympus Plan Apo 69×, NA 1.42 oil immersion objective and a CoolSNAP HQ2 charge-coupled device camera (Photometrics, Tucson, AZ, USA). Fixed cells were imaged using an Olympus IX70 microscope comprised of an Olympus Plan Apo 60×, NA 1.4 oil immersion objective and a CoolSNAP HQ camera (Roper Scientific, Netherlands). Images were captured in 0.4 µm increments through the *z*-axis in stacks of 10 or 12 images. All image acquisition and subsequent deconvolution were performed using SoftWorx DeltaVision software (GE Healthcare). Z-stacks were deconvolved and then combined to form a two-dimensional projection using a maximum intensity algorithm. Images were analysed in Fiji (NIH) and processed with Adobe Photoshop version CS5.1 or CC 2018 and Adobe Illustrator CS5.1 or CC 2018. For live-cell time-lapse imaging, log-phase cultures grown at 30°C were shifted to 36°C for 40 min prior to imaging at 36°C. Live cells were adhered to a glass-bottomed culture dish (MatTek Corporation, Ashland, MA, USA) using soybean lectin and covered with warmed YE5S media. An AirTherm SMT temperature-controlled chamber (World Precision Instruments, Sarasota, FL, USA) maintained cells at 36°C during time-lapse imaging. Fixed cells were imaged on glass slides at room temperature. Cells were fixed using cold methanol and acetone and resuspended in PBS, followed by staining for DNA with 4′,6-diamidine-2′-phenylindole (DAPI; Vectashield, Vector Laboratories, Burlingame, CA, USA).

### Immunochemistry

Protein extracts were prepared from cells subjected to mechanical lysis. Briefly, cells were suspended in IP buffer [50 mM Tris­HCl pH 7.4, 1 mM EDTA, 150 mM NaCl, 0.05% NP-40 (IGEPAL CA630; Sigma-Aldrich), 10% (v/v) glycerol] supplemented with 1 mM DTT, 15 mM *p*-nitrophenyl phosphate, 1 mM phenylmethlysulfonyl fluoride and protease inhibitor cocktail (Sigma-Aldrich) before beating with acid-washed beads at 4°C using a FastPrep FP120 cell disrupter (BIO101; Thermo Savant). Debris was then cleared from extracts by centrifugation for 1 min followed by a further 5 min at 13,000 rpm. Protein concentration was determined by Bradford assay (Bio-Rad Laboratories, Hercules, CA, USA).

For immunoprecipitation, whole cell extracts (WCEs) were collected and Klp6-GFP precipitated out of the extracts using the magnetic GFP-trap system (ChromoTek, Martinsried, Germany), where anti-GFP VHH antibody is coupled to magnetic beads. Protein extract (2.8 mg) was incubated with GFP-trap for 1.5 h at 4°C with rotation. The immunocomplexed beads were then washed with and resuspended in immunoprecipitation buffer before boiling in Laemmli buffer for 5 min to separate immunocomplexes from beads. WCEs or immunocomplexes were then separated by SDS-PAGE and analysed by immunoblotting.

For both immunoprecipitation of Klp5-5FLAG with Klp6-GFP and for checking the expression levels of Klp5-5FLAG constructs by Western blot, immunoblotting was performed as follows: Klp5 was detected using mouse anti-FLAG M2 antibody (F-3165; Sigma-Aldrich) at a dilution of 1:1000 and Klp6 was detected by rabbit anti-GFP (TP401; AMS Biotechnology, Abingdon, UK) at a dilution of 1:1000, followed by incubation with secondary anti-mouse or anti-rabbit antibodies conjugated to horseradish peroxidase (GE Healthcare). The ECL chemiluminescence kit (GE Healthcare) was used to detect signals.

### Protein expression and purification

A pET-Duet plasmid (Novagen; Merck, Darmstadt, Germany) containing codon-optimised *klp5­HA­PreScission site-proteinA* and *klp6­msfGFP* was used to express WT proteins and as a template for mutagenesis. For the study of Klp5^WT/Q582P^-HA/Klp6­msfGFP, *klp5­HA-PreScission site­proteinA* was mutated to *klp5^Q582P^* using the In­Fusion HD Cloning Kit (Clontech Laboratories, Takara Bio, Shiga, Japan). Kinesin-8 constructs were co-expressed in *E. coli* BL21-Codon-Plus(DE3)-RIL cells (Agilent Technologies) grown at 37°C then induced with 0.1 mM IPTG at 18°C for 16 h. Cells were harvested in ice-cold LEW+ (lysis-equilibration-wash buffer plus protease inhibitors and DNase) buffer [50 mM HEPES pH 7.5, 300 mM KCl, 2 mM MgCl_2_, 0.002% Brij-35, 1 mM DTT, 10% (v/v) glycerol, 0.2 mM ATP], plus a cocktail of EDTA-free protease inhibitors (Roche, Basel, Switzerland) and DNase I (Sigma-Aldrich). Cells were lysed by passing the suspension twice through a chilled high-pressure homogeniser (Emulsiflex C5, Microfluidics; ATA Scientific, Taren Point, NSW, Australia). Samples were then passed through a sieve and clarified by centrifugation at 50,000 rpm for 40 min at 4°C to pellet debris. For purification of the kinesin-8 complex, lysates were first incubated with IgG Sepharose­6 Fast­Flow resin (GE Healthcare) for 2 h at 4°C. The resin was then transferred into a LEW+-equilibrated Econo-Pac column (Bio-Rad Laboratories, Hercules, CA, USA) and washed with 10 column volumes of LEW+ buffer to remove unbound protein. To cleave the Protein­A tag from Klp5-HA, samples were incubated at 4°C overnight in LEW buffer containing a PreScission protease (GE Healthcare). Cleavage was checked by SDS-PAGE and Coomassie Brilliant Blue staining before proteins were taken for further purification by gel filtration. A Superose-6 10/330 column was equilibrated with LEW buffer at 4°C and used to purify the Klp5-HA/Klp6-sfGFP complexes, during which proteins eluted as a large majority peak, suggestive of a homogenous population of heterodimers. Purified proteins were then flash-frozen in, and subsequently stored in, liquid nitrogen until required.

Porcine brain tubulin was purified and cycled tubulin fractions were labelled with Cy5 (cyanin5 NHS ester; Lumiprobe, Hunt Valley, MD, USA) or EZ-link NHS Biotin (ThermoFisher Scientific, Waltham, MA, USA) as described previously (Bieling et al., 2007). Protein concentrations were determined by Bradford assay (Bio-Rad Laboratories).

### TIRF-M and image analysis

TIRF-M dynamic MT assays were performed as previously described (Bieling et al., 2010). Flow chambers were assembled using a coverslip functionalised with biotin-PEG attached to a PLL-PEG passivated microscope slide via double-sided tape. For imaging of GMPCPP-stabilised MTs, dim Cy5-labelled MTs were polymerised from MT seeds stabilised with glass-immobilised brightly labelled (33% Cy5-labelled tubulin) GMPCPP (Jena Bioscience, Jena, Germany) in the presence of Klp5-Klp6 complex. For imaging of MTs stabilised with taxol (Paclitaxel, 33069-62-4; Sigma-Aldrich) or GMPCPP, the assay buffer consisted of BRB80 supplemented with 1 mM ATP,1 mM GTP, 10 mM β-mercaptoethanol, 0.1% Brij-35, 0.1% methylcellulose (4000 centipoise; Sigma-Aldrich) and an oxygen scavenger system [20 mM glucose, 320 µg/ml glucose oxidase (Serva Electrophoresis, Heidelberg, Germany) and 55 µg/ml catalase (Sigma-Aldrich)]. For imaging of dynamic MTs, the assay buffer contained 85 mM KCl and 85 mM CH_3_COOK and the oxygen scavenger system was used. All experiments were performed at 30°C.

Simultaneous dual-colour time-lapse imaging of Cy5-labelled MTs and GFP-tagged kinesin-8 was performed on a custom TIRF microscope using a 100× objective equipped with a Cascade II cooled charge-coupled device camera (Photometrics, Tucson, AZ, USA) at 30±1°C. Images were acquired at 1 s intervals with an exposure time of 100 ms, using 488 nm and 640 nm lasers to illuminate the slide.

Images were analysed using ImageJ software (NIH) and various custom macros. A bleach correcting macro (corr_bleach050405; J. Rietdorf, EMBL, Heidelberg, Germany) was applied to separate channels before merging to create a composite image. An image stabiliser macro (Image_stabilizer.class, K. Li, ‘The image stabilizer plugin for ImageJ’, http://www.cs.cmu.edu/~kangli/code/Image_Stabilizer.html, February, 2008) was applied to correct for field drift. Dynamic MT growth rate was calculated by measuring the increase in MT length over time. MT catastrophe frequency was determined for a single growing MT by dividing the number of catastrophe events observed by the total duration of the growth period. To characterise GFP-tagged kinesin-8 behaviour on taxol-stabilised MTs, kymographs were generated for each MT and run events of kinesin-8 complex were analysed. Processive runs were classed as those showing smooth, constant and directed movement, which also allowed assignment of the plus end because WT kinesin-8 is plus-end-directed. Diffusive runs were characterised by stochastic back and forth continued movement on the MT. Note that diffusive runs exhibited an overall directionality for the plus end. In the event that the GFP signal bound to the MT but did not display any movement over time, the run was classified as static. Only runs proceeding for 3 s or more (three pixels) were included for characterisation.

### Statistical analysis

In all experiments where statistical data are presented, sample sizes are given as numbers next to each data set on graphs. Statistical evaluation was performed using Prism (GraphPad Software, San Diego, CA, USA). All *P*-values were from two-tailed Mann–Whitney tests, unless otherwise stated.

## Supplementary Material

Supplementary information
