# Supplementary material for: Kinesin-8 and Dis1/TOG collaborate to limit spindle elongation from prophase to anaphase A for proper chromosome segregation in fission yeast
Source: J Cell Sci. 2019 Sep 23;132(18):jcs232306. doi: 10.1242/jcs.232306 (PMC6765184; doi:10.1242/jcs.232306)
Supplement: Supplementary information [file joces-132-232306-s1.pdf]

Supplementary Information

Table S1. List of fission yeast strains used in this study

Strain	Genotype	Source
	**Figure 1**	
CP012	*h+ leu1-32 ura4-D18 his2 klp5::kanMX6*	Lab stock
CP024	*h- leu1-32 ura4-D18 dis1::hphMX6*	Lab stock
CP002	*h- leu1-32 ura4-D18 513*	Lab stock
CP102	*h- leu1-32 ura4-D18 klp5^{CP76}-5FLAG-kanMX6*	This study
CP118	*h- leu1-32 ura4-D18 dis1::hphMX6 klp5-5FLAG-kanMX6*	This study
CP196	*h- leu1-32 ura4-D18 klp5^{Q582P}-5FLAG-kanMX6*	This study
CP017	*h^{-} leu1-32 ura4-D18 klp5-5FLAG-kanMX6*	Lab stock
CP021	*h- leu1-32 ura4-D18 klp6-GFP-kanMX6*	Lab stock
CP521	*h? leu1-32 ura4-D18 klp5-5FLAG-kanMX6 dis1::hphMX6 klp6-GFP-kanMX6*	This study
CP508	*h- leu1-32 ura4-D18 klp5^{Q582P}-5FLAG-kanMX6 dis1::hphMX6 klp6-GFP-kanMX6*	This study
	**Figure 2**	
CP352	*h- leu1-32 ura4-D18 cen2:hphMX6-lacOp his7:lacI-GFP-ura4+ aur1C-mCherry-atb2 sad1-dsRED-LEU2*	This study
CP473	*h- leu1-32 ura4-D18 klp6::kanMX6 cen2:hphMX6-lacOp his7:lacI-GFP-ura4+ aur1C-mCherry-atb2 sad1-dsRED-LEU2*	This study
CP587	*h- leu1-32 ura4-D18 klp5^{Q582P}-5FLAG-kanMX6 cen2:hphMX6-lacOp his7:lacI-GFP-ura4+ aur1C-mCherry-atb2 sad1-dsRED-LEU2*	This study
CP002	*h- leu1-32 ura4-D18 513*	Lab stock
CP009	*h- leu1-32 ura4-D18 klp5::kanMX6*	Lab stock
CP041	*h- leu1-32 ura4-D18 his2/7 klp6::kanMX6*	Lab stock
CP052	*h+ leu1-32 ura4-D18 his2/7 klp5::ura4+ klp6::kanMX6*	This study
CP196	*h- leu1-32 ura4-D18 klp5^{Q582P}-5FLAG-kanMX6*	This study
CP778	*h^{-} leu1-32 ura4-D18 klp5^{E575P}-5FLAG-kanMX6*	This study
CP850	*h^{-} leu1-32 ura4-D18 klp6^{E569P}-5FLAG-kanMX6*	This study
CP851	*h^{-} leu1-32 ura4-D18 klp5^{E575P}-5FLAG-kanMX6 klp6^{E569P}-5FLAG-kanMX6*	This study
CP852	*h^{-} leu1-32 ura4-D18 klp5^{E575P}-5FLAG-kanMX6 klp6^{E569P}-5FLAG-kanMX6*	This study
CP024	*h- leu1-32 ura4-D18 dis1::hphMX6*	Lab stock

Journal of Cell Science • Supplementary information

Figure 5

CP180	h+ leu1-32 ura4-D18 his2 nuf2-YFP-ura4+ sid4-mRFP-natMX6 aur1C-mCherry-atb2	Lab stock
CP239	h+ leu1-32 ura4-D18 his2 dis1::hphMX6 nuf2-YFP-ura4+ sid4-mRFP-natMX6 aur1C-mCherry-atb2	This study
CP324	h+ leu1-32 ura4-D18 his2 klp5::kanMX6 nuf2-YFP-ura4+ sid4-mRFP-natMX6 aur1C-mCherry-atb2	This study
CP317	h- leu1-32 ura4-D18 klp5^{Q582P}-5FLAG-kanMX6 dis1::hphMX6 nuf2-YFP-ura4+ sid4-mRFP-natMX6 aur1C-mCherry-atb2	This study

Figure 6

CP180	h+ leu1-32 ura4-D18 his2 nuf2-YFP-ura4+ sid4-mRFP-natMX6 aur1C-mCherry-atb2	Lab stock
CP239	h+ leu1-32 ura4-D18 his2 dis1::hphMX6 nuf2-YFP-ura4+ sid4-mRFP-natMX6 aur1C-mCherry-atb2	This study
CP324	h+ leu1-32 ura4-D18 his2 klp5::kanMX6 nuf2-YFP-ura4+ sid4-mRFP-natMX6 aur1C-mCherry-atb2	This study
CP317	h- leu1-32 ura4-D18 klp5^{Q582P}-5FLAG-kanMX6 dis1::hphMX6 nuf2-YFP-ura4+ sid4-mRFP-natMX6 aur1C-mCherry-atb2	This study

Figure 7

CP180	h+ leu1-32 ura4-D18 his2 nuf2-YFP-ura4+ sid4-mRFP-natMX6 aur1C-mCherry-atb2	Lab stock
CP239	h+ leu1-32 ura4-D18 his2 dis1::hphMX6 nuf2-YFP-ura4+ sid4-mRFP-natMX6 aur1C-mCherry-atb2	This study
CP324	h+ leu1-32 ura4-D18 his2 klp5::kanMX6 nuf2-YFP-ura4+ sid4-mRFP-natMX6 aur1C-mCherry-atb2	This study
CP317	h- leu1-32 ura4-D18 klp5^{Q582P}-5FLAG-kanMX6 dis1::hphMX6 nuf2-YFP-ura4+ sid4-mRFP-natMX6 aur1C-mCherry-atb2	This study
CP818	h- leu1-32 ura4-D18 klp9-YFP-natMX6 hht1-CFP-natMX6 aur1C-mCherry-atb2	This study
CP820	h- leu1-32 ura4-D18 klp5::ura4+ klp9-YFP-natMX6 hht1-CFP-natMX6 aur1C-mCherry-atb2	This study
CP831	h- leu1-32 ura4-D18 dis1::hphMX6 klp5^{Q582P}-5FLAG-kanMX6 klp9-YFP-natMX6 dis1::hphMX6 hht1-CFP-natMX6 aur1C-mCherry-atb2	This study
CP557	h- leu1-32 ura4-D18 klp5^{Q582P}-5FLAG-kanMX6 dis1::hphMX6 cut11-GFP-ura4+ sid4-mRFP-natMX6 aur1C-mCherry-atb2	This study

Supplementary Figure S1

CP017	h$^-$ leu1-32 ura4-D18 klp5-5FLAG-kanMX6	Lab stock
CP026	h- leu1-32 ura4-D18 klp5-5FLAG-kanMX6 dis1::ura4+	This study
CP118	h- leu1-32 ura4-D18 dis1::hphMX6 klp5^{CP76}-5FLAG-kanMX6	This study
CP002	h- leu1-32 ura4-D18 513	Lab stock
CP197	h$^-$ leu1-32 ura4-D18 dis1::hphMX6 klp5^{Q582P}-5FLAG-kanMX6	This study
CP295	h- leu1-32 ura4-D18 dis1::hphMX6 klp5^{D843G}-5FLAG-kanMX6	This study

Journal of Cell Science • Supplementary information

Supplementary Figure S2

CP558	*h- leu1-32 ura4-D18 klp5::ura4+ cut11-GFP-ura4+ sid4-mRFP-natMX6 aur1C-mCherry-atb2*	This study
CP902	*h- leu1-32 ura4-D18 klp6::ura4+ cut11-GFP-ura4+ sid4-mRFP-natMX6 aur1C-mCherry-atb2*	This study
CP554	*h- leu1-32 ura4-D18 cut11-GFP-ura4+ sid4-mRFP-natMX6 aur1C-mCherry-atb2*	This study
CP555	*h- leu1-32 ura4-D18 klp5*^{Q582P}*-5FLAG-kanMX6 cut11-GFP-ura4+ sid4-mRFP-natMX6 aur1C-mCherry-atb2*	This study
CP793	*h- leu1-32 ura4-D18 klp5*^{E575P}*-5FLAG-kanMX6 cut11-GFP-ura4+ sid4-mRFP-natMX6 aur1C-mCherry-atb2*	This study

Supplementary Figure S5

CP180	*h+ leu1-32 ura4-D18 his2 nuf2-YFP-ura4+ sid4-mRFP-natMX6 aur1C-mCherry-atb2*	Lab stock
CP239	*h+ leu1-32 ura4-D18 his2 dis1::hphMX6 nuf2-YFP-ura4+ sid4-mRFP-natMX6 aur1C-mCherry-atb2*	This study
CP324	*h+ leu1-32 ura4-D18 his2 klp5::kanMX6 nuf2-YFP-ura4+ sid4-mRFP-natMX6 aur1C-mCherry-atb2*	This study
CP317	*h- leu1-32 ura4-D18 klp5*^{Q582P}*-5FLAG-kanMX6 dis1::hphMX6 nuf2-YFP-ura4+ sid4-mRFP-natMX6 aur1C-mCherry-atb2*	This study

Supplementary Figure S6

CP544	*h+ leu1-32? ura4-D18? his2? klp5*^{Q582P}*-5FLAG-kanMX6 dis1::hphMX6 cdc13-sfGFPcp sid4-mRFP-natMX6 aur1C-mCherry-atb2*	This study
CP901	*h leu1-32 ura4-D18 klp5*^{Q582P}*-5FLAG-kanMX6 dis1::hphMX6 pmo25-GFP-kanMX6 Sid4-mRFP-natMX6*	This study

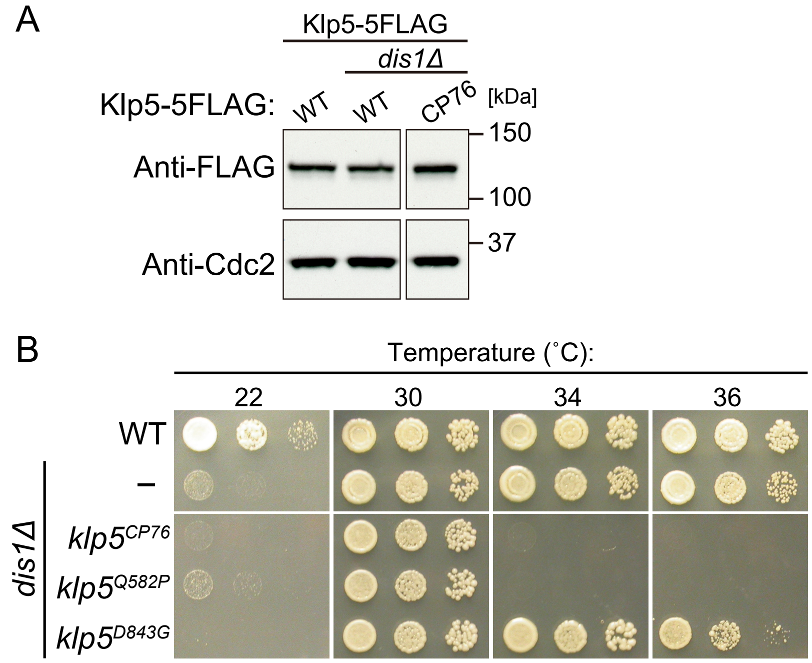

Figure S1. Klp5^{CP76} is a full length mutant protein in which the Q582P substitution is responsible for temperature-sensitivity in the *dis1Δ* background

(A) Cells were shifted to 36˚C for 1 hour. Lysates were analysed by SDS-PAGE and Western blotting using anti-FLAG or anti-Cdc2 antibodies (loading control). **(B)** Serial dilution assay. YE5S plates were incubated at the indicated temperatures for 3 days.

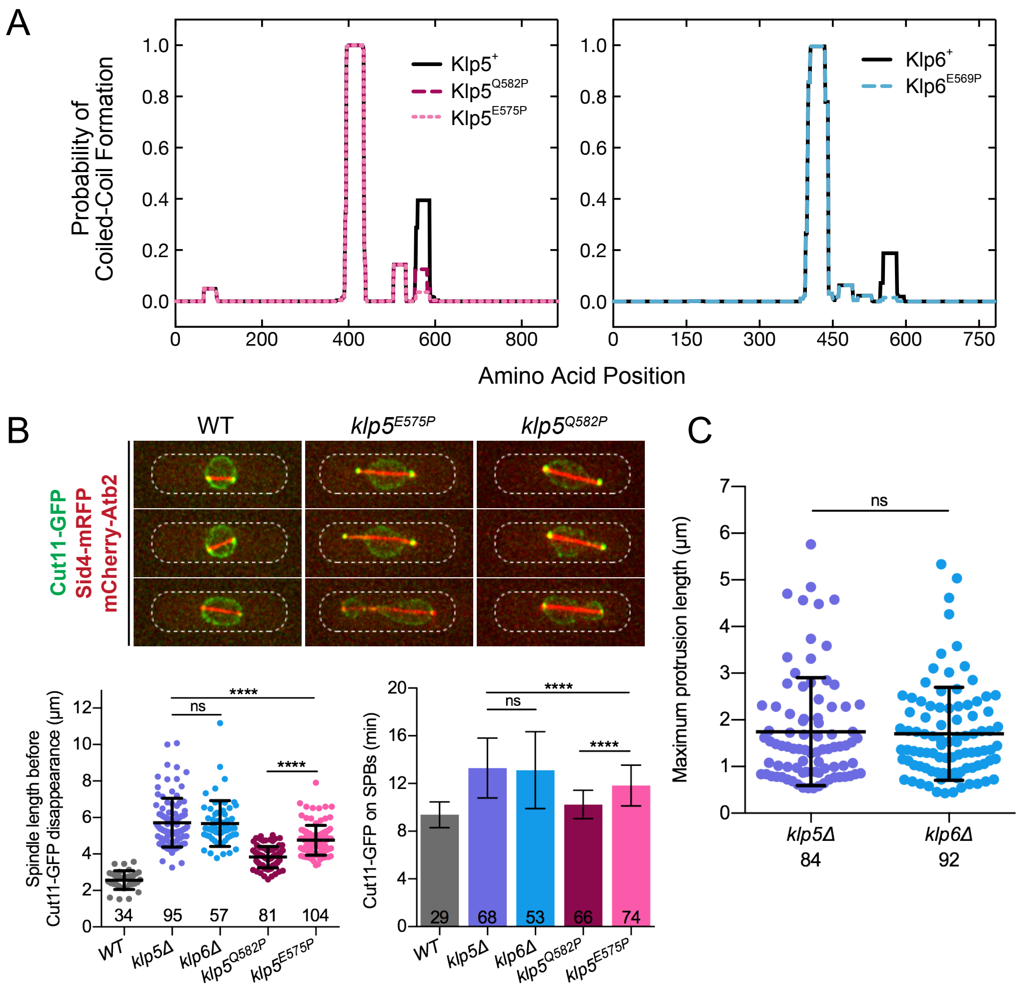

Figure S2. The tailbox is a functional motif in both Klp5 and Klp6

(A) Graphs depicting probability of CC formation for kinesin-8 proteins using protein sequence (x-axis; amino acid position). Prediction performed using COILS (Lupas et al., 1991) prediction software on MTIDK matrix using window 28 with a weight of 2.5 at heptad positions a and d. Left - Solid black trace - WT Klp5 protein sequence; dashed magenta trace - Klp5^{Q582P} protein sequence; dotted pink trace - Klp5^{E575P} protein sequence. Right - Solid black trace - WT Klp6 protein sequence; dashed blue trace - Klp6^{E569P} protein sequence. **(B)** Left - representative time-lapse images of WT, *klp5^{E575P}* and *klp5^{Q582P}* cells expressing the nuclear envelope marker Cut11-GFP (green) (West et al., 1998) along with an SPB marker Sid4-mRFP (Chang and Gould, 2000) and the tubulin marker mCherry-Atb2 (red). Images were acquired at 2 minute intervals at 36˚C. Scale bar, 10 µm. Middle –spindle length before Cut11-GFP disappearance in WT, *klp5Δ*, *klp6Δ*, *klp5^{Q582P}* and *klp5^{E575P}* cells. ****, p<0.0001; ns, p=0.9856. Right - mean duration of Cut11-GFP signal on SPBs in WT, *klp5Δ*, *klp6Δ*, *klp5^{Q582P}* and *klp5^{E575P}* cells. Error bars represent mean and standard deviation, and the number of samples analysed is shown. ****, p<0.0001; ns, p=0.5930. **(C)** Maximum spindle protrusion lengths for pre-anaphase *klp5Δ* and *klp6Δ* cells at 36˚C. ns, p=0.7920.

Journal of Cell Science • Supplementary information

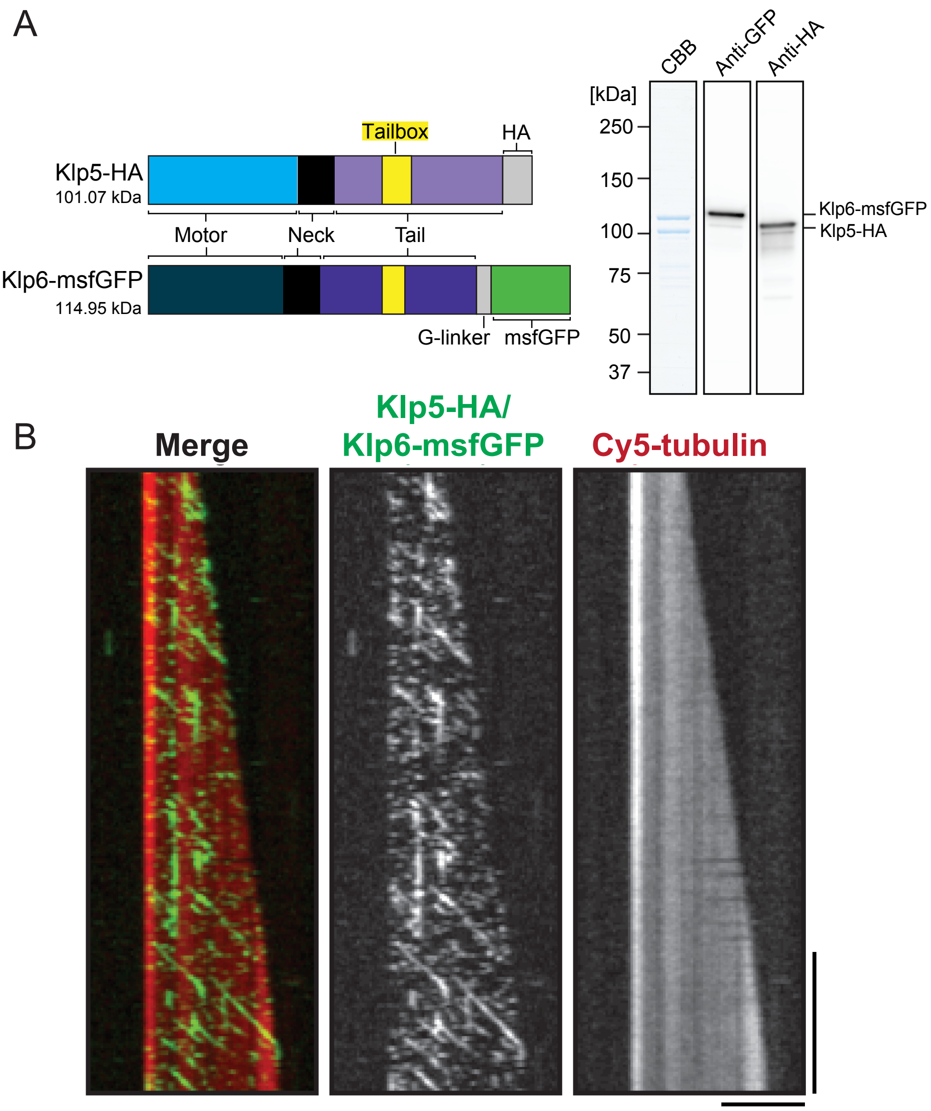

Figure S3. Full-length Klp5-Klp6 complex is purified to a high degree and shows plus-end directed motility on dynamic MTs *in vitro*

(A) Schematic of fluorescently-tagged Klp5-HA/Klp6-msfGFP protein constructs co-expressed and purified; recombinant proteins were analysed by SDS-PAGE and detected by either Coomassie Brilliant Blue staining (CBB, left) or by Western blotting using anti-GFP (middle) or anti-HA (right) antibodies to detect Klp6-msfGFP and Klp5-HA, respectively. **(B)** Representative dual-colour TIRF-M kymographs depicting growing Cy5-labelled MTs (red) with 10 nM Klp5-HA/Klp6-msfGFP (green) in the presence of 10 µM Cy5-labelled tubulin and 1 mM ATP. Vertical scale bar, 1 min; horizontal scale bar, 5 µm.

Journal of Cell Science • Supplementary information

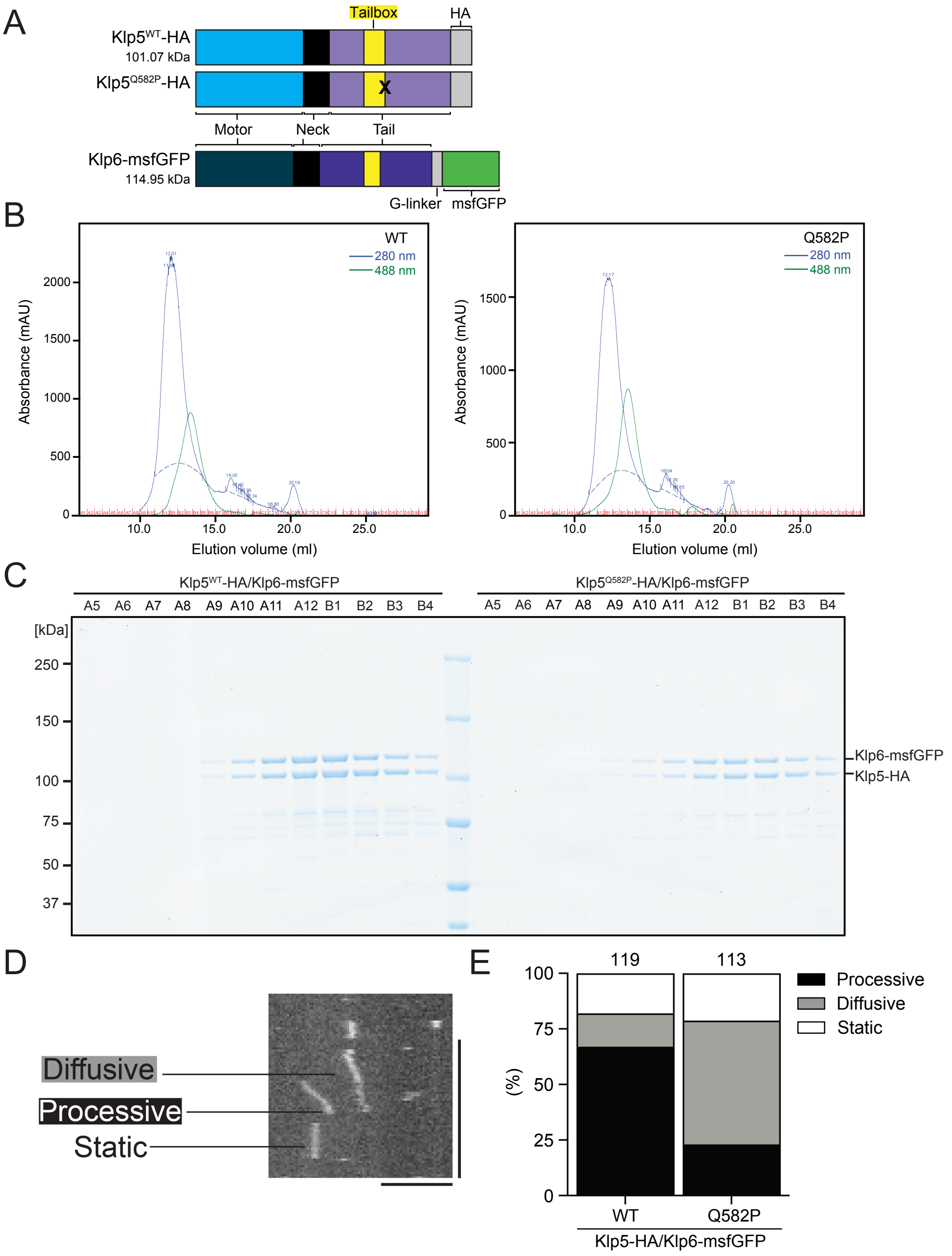

Figure S4. The recombinant Klp5^{Q582P}-HA/Klp6-msfGFP complex is properly folded and assembled as in the Klp5^{WT}-HA/Klp6-msfGFP complex

(A) Schematic of fluorescently-tagged WT and TB mutant Klp5-HA/Klp6-msfGFP protein constructs co-expressed and purified. **(B)** Gel filtration chromatograms of Klp5^{WT}-HA/Klp6-msfGFP (left) and Klp5^{Q582P}-HA/Klp6-msfGFP (right). Solid lines represent sample absorbance at 280 nm (blue) and 488 nm (green). Fractions are labelled in red. The peak positions of 288nm and 488nm do not overlap completely, the reason for which is currently unexplored. **(C)** Appropriate gel filtration fractions were collected, analysed by SDS-PAGE and detected by CBB staining. M, molecular weight standards – positions represented on right and corresponding CBB patterns shown in middle lane. **(D)** Examples of run types assigned in Figs. 4B and S4E. **(E)** Categories of fluorescently-tagged kinesin-8 binding event to taxol-stabilised MTs. Frequency of processive (black), diffusive (grey) or static (white) kinesin-8 runs by the WT or Q582P mutant complex is graphed. The number of samples analysed is shown.

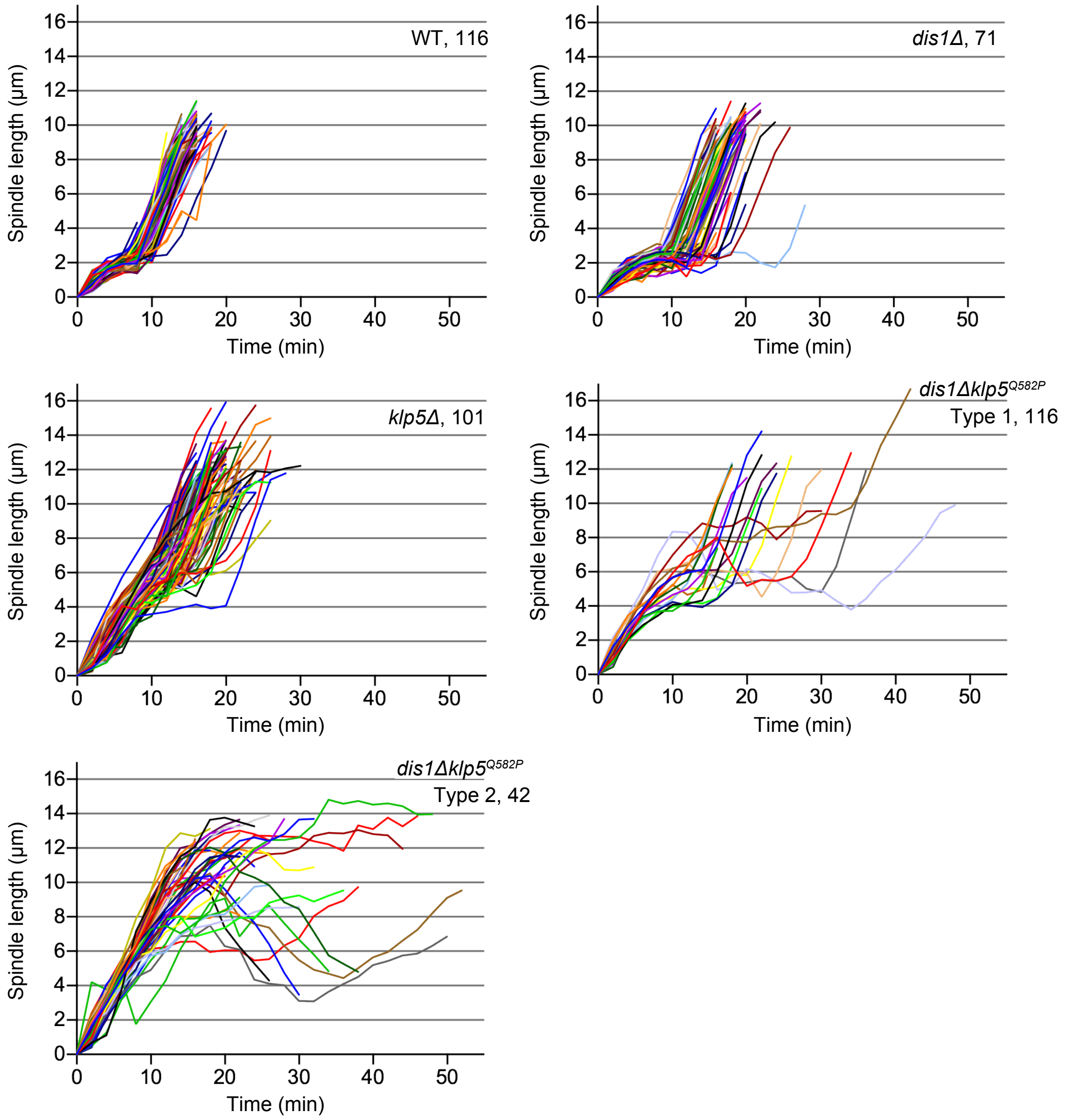

Figure S5. Kinesin-8 and Dis1 are both required for normal changes in mitotic spindle length

Data comes from experiment presented in Fig. 4. *dis1Δklp5^{Q582P}* cells show two types of spindle behaviour. Mitotic spindle lengths were plotted against time for WT, *dis1Δ*, *klp5Δ*, type 1 and type 2 *dis1Δklp5^{Q582P}* cells. The number of samples analysed is shown.

Journal of Cell Science • Supplementary information

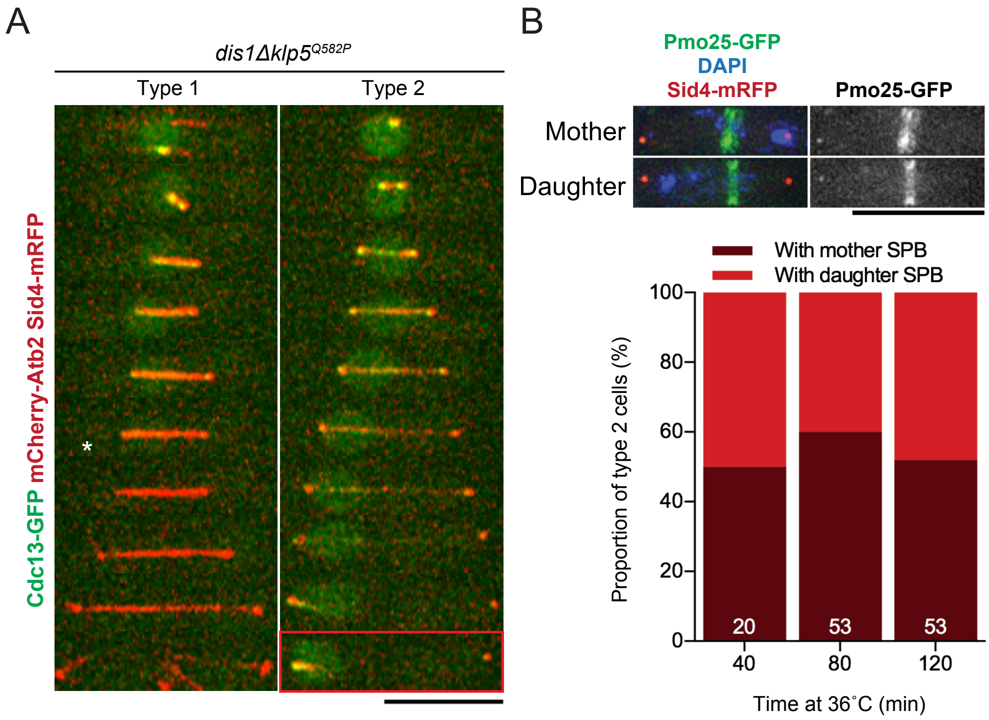

Figure S6. Terminal phenotypes of *dis1Δklp5*^{Q582P} cells

(A) Long spindles are pre-anaphase. Representative kymographic images of Cdc13-sfGFPcp (green) (Kamenz et al., 2015) in type 1 and type 2 *dis1Δklp5*^{Q582P} cells also expressing the SPB marker Sid4-mRFP and tubulin marker mCherry-Atb2 (red). Images were acquired at 2 minute intervals at 36˚C. Asterisk indicates disappearance of Cdc13-sfGFPcp signal. Frame outlined in red is 10 minutes later than the previous frame. **(B)** Exponentially growing cultures of *dis1Δ klp5*^{Q582P} cells expressing Pmo25-GFP along with Sid4-mRFP were shifted to 36˚C then fixed and DNA was stained by DAPI. Left - representative type 2 cells are shown. Right – type 2 cells were scored by the identity of the SPB. The number of samples analysed is shown. Scale bars, 10 µm.

Journal of Cell Science • Supplementary information

SUPPLEMENTARY References

Chang, L. and Gould, K. L. (2000). Sid4p is required to localize components of the septation initiation pathway to the spindle pole body in fission yeast. *Proc Natl Acad Sci USA*. **97**, 5249-5254.

Kamenz, J., Mihaljev, T., Kubis, A., Legewie, S. and Hauf, S. (2015). Robust ordering of anaphase events by adaptive thresholds and competing degradation pathways. *Mol Cell*. **60,** 446-459.

Lupas, A., Van Dyke, M. and Stock, J. (1991). Predicting coiled coils from protein sequences. *Science*. **252**, 1162-1164.

West, R. R., Vaisberg, E. V., Ding, R., Nurse, P. and McIntosh, J. R. (1998). *cut11*$^{+}$: A gene required for cell cycle-dependent spindle pole body anchoring in the nuclear envelope and bipolar spindle formation in *Schizosaccharomyces pombe*. *Mol Biol Cell*. **9**, 2839-2855.

Journal of Cell Science • Supplementary information
